# Nabiximols combined with motivational enhancement/cognitive behavioral therapy for the treatment of cannabis dependence: A pilot randomized clinical trial

**DOI:** 10.1371/journal.pone.0190768

**Published:** 2018-01-31

**Authors:** Jose M. Trigo, Alexandra Soliman, Lena C. Quilty, Benedikt Fischer, Jürgen Rehm, Peter Selby, Allan J. Barnes, Marilyn A. Huestis, Tony P. George, David L. Streiner, Gregory Staios, Bernard Le Foll

**Affiliations:** 1 Translational Addiction Research Laboratory, Campbell Family Mental Health Research Institute, Centre for Addiction and Mental Health (CAMH), Toronto, Canada; 2 Campbell Family Mental Health Research Institute, CAMH, Toronto, Canada; 3 Department of Psychiatry, University of Toronto, Toronto, Canada; 4 Institute of Medical Science, University of Toronto, Faculty of Medicine, Toronto, Canada; 5 Institute for Mental Health Policy Research, CAMH, Toronto, Canada; 6 Centre for Applied Research in Mental Health & Addiction, Faculty of Health Sciences, Simon Fraser University, Vancouver, Canada; 7 Addiction Policy, Dalla Lana School of Public Health, University of Toronto, Toronto, Canada; 8 Institute of Clinical Psychology and Psychotherapy & Center of Clinical Epidemiology and Longitudinal Studies (CELOS), Technische Universität Dresden, Dresden, Germany; 9 Addictions Division, CAMH, Toronto, Canada; 10 Department of Family and Community Medicine, University of Toronto, Toronto, Canada; 11 Chemistry and Drug Metabolism, National Institute on Drug Abuse (NIDA), National Institutes of Health (NIH), Baltimore, United States of America; 12 Division of Brain and Therapeutics, CAMH, Toronto, Canada; 13 Department of Psychiatry & Behavioural Neurosciences, McMaster University, Hamilton, Canada; Columbia University, UNITED STATES

## Abstract

**Background:**

The current lack of pharmacological treatments for cannabis use disorder (CUD) warrants novel approaches and further investigation of promising pharmacotherapy. We previously showed that nabiximols (27 mg/ml Δ^9^-tetrahydrocannabinol (THC)/ 25 mg/ml cannabidiol (CBD), Sativex^®^) can decrease cannabis withdrawal symptoms. Here, we assessed in a pilot study the tolerability and safety of self-titrated nabiximols vs. placebo among 40 treatment-seeking cannabis-dependent participants.

**Methods:**

Subjects participated in a double blind randomized clinical trial, with as-needed nabiximols up to 113.4 mg THC/105 mg CBD or placebo daily for 12 weeks, concurrently with Motivational Enhancement Therapy and Cognitive Behavioral Therapy (MET/CBT). Primary outcome measures were tolerability and abstinence, secondary outcome measures were days and amount of cannabis use, withdrawal, and craving scores. Participants received up to CDN$ 855 in compensation for their time.

**Results:**

Medication was well tolerated and no serious adverse events (SAEs) were observed. Rates of adverse events did not differ between treatment arms (F_1,39_ = 0.205, NS). There was no significant change in abstinence rates at trial end. Participants were not able to differentiate between subjective effects associated with nabiximols or placebo treatments (F_1,40_ = 0.585, NS). Cannabis use was reduced in the nabiximols (70.5%) and placebo groups (42.6%). Nabiximols reduced cannabis craving but no significant differences between the nabiximols and placebo groups were observed on withdrawal scores.

**Conclusions:**

Nabiximols in combination with MET/CBT was well tolerated and allowed for reduction of cannabis use. Future clinical trials should explore the potential of high doses of nabiximols for cannabis dependence.

## Introduction

Cannabis is the most widely used illicit substance worldwide [1]. Those who have used cannabis at least once (aged 15 to 64) are estimated to be 128–232 million, or 2.7 to 4.9% of the world’s population [2]. There is a high prevalence of use in North America and a gradual increase since 2007 [2]. Cannabis use prevalence has important implications for public health [3–5] and its use has been associated with a variety of health problems including cognitive [6] and respiratory impairment [7], psychotic episodes [8], injury risk [9] and dependence [10,11]. Research indicates that about 8% of those who ever use cannabis may develop cannabis dependence [12,13]. However, there is currently no approved pharmacotherapy for cannabis dependence [14,15]. Due to the significant impact of problematic cannabis use on individuals and society, and thereby, the increasing demand for treatment, several research teams have focused on developing medications for cannabis dependence treatment [14,16]. These studies have mainly tested the potential utility of pharmacotherapies available for other indications (e.g. cannabinoid drugs, antidepressants, anxiolytics, and antipsychotics). Reviews indicated that Δ^9^-tetrahydrocannabinol (THC), the anticonvulsant gabapentin and the glutamatergic modulator N-acetylcysteine (clinical trials # NCT00974376 and NCT01675661, respectively), are the most promising approaches [14,17,18]. Although recent studies have also shown that N-acetylcysteine might have limited effects in adults [19]. The potential benefits of cannabinoid agonist preparations like THC or the synthetic analogs Nabilone and Dronabinol for cannabis dependence treatment have been evaluated in several studies [20–26]. Though these THC and THC-analogs showed promising effects on cannabis withdrawal symptoms, they did not reduce cannabis use in some of these laboratory studies [21,23]. On the other hand, more recent studies have shown that participants used less cannabis while maintained on Nabilone [26] or a combination of Nabilone and Zolpidem [27] under laboratory conditions.

Preclinical studies suggest that cannabidiol (CBD) might modulate neuronal circuits involved in drug addiction, featuring the potential to reduce addiction (see [28] review). Recently, there is growing interest in the ~1:1 THC/CBD combination (also called nabiximols or Sativex^®^ [brand name]) for cannabis dependence treatment [29]. This ~1:1 THC/CBD combination (developed by GW Pharma) was approved for multiple sclerosis treatment in humans in several European countries and Canada. Recently, we showed that nabiximols is effective to alleviate cannabis withdrawal [30]. In addition, Allsop *et al*. tested nabiximols in Australian treatment-seeking participants with cannabis dependence showing reduction in cannabis withdrawal scores and improved treatment retention but no reduction in cannabis use compared to placebo [31]. However, pharmacological treatment was limited to six days of exposure and participants were treated initially in an inpatient unit, so the trial was limited in its ability to test efficacy for long-term cannabis use or abstinence. Therefore, there is a great need to explore the impact of prolonged administration of THC/CBD combinations in treatment-seeking participants with cannabis dependence.

In the present study, we explored the effects of a three month course of self-titrated nabiximols treatment combined with Motivational Enhancement Therapy and Cognitive Behavioral Therapy (MET/CBT) on cannabis dependence. The main objective of this pilot study was to determine if the self-titrated dosage was well tolerated and sufficient to observe any effects on cannabis use, craving and withdrawal in comparison with placebo.

## Materials and methods

### Study design

The study was a double-blind, placebo-controlled randomized clinical trial in which 40 participants underwent an experimental procedure consisting of a 12-week course of treatment (self-titration of placebo or nabiximols, up to a maximum of 42 sprays, equal to 113.4 mg THC/105 mg CBD daily) and MET/CBT (S1 Fig). The trial was approved by the Centre for Addiction and Mental Health (CAMH) Research Ethics Board (Protocol #144/2011) and was authorized by Health Canada. It was conducted in compliance with ICH E6: Good Clinical Practice and applicable Canadian regulatory requirements. Written informed consent was obtained from the participants. The study was registered on Clinical Trials.gov (NCT 01747850).

### Participants

Inclusion criteria were a) 18–65 year old male or female; b) understanding and willingness to comply with study requirements and restrictions; c) willingness to use appropriate contraceptive method throughout the study; d) physical health based on medical history, physical exam, vitals, ECG and chemistry and hematological laboratory results; e) meet DSM-IV criteria for current cannabis dependence; f) report cannabis as primary drug of abuse; g) report using cannabis at least 5 days a week for at least one month; h) have cannabinoid positive urine drug screen; i) treatment-seeking for cannabis dependence; and j) smoke less than or equal to the equivalent of 4 joints per day (or four grams per day if participants smokes cannabis in other forms).

Exclusion criteria were a) meeting DSM-IV criteria for a current Axis I disorder including substance use disorder other than cannabis, nicotine or caffeine dependence; b) having a first-degree relative with schizophrenia; c) history of seizures; d) history of cardiovascular disease; e) history of pulmonary disease such as asthma or COPD; f) clinically significant pathology in oral cavity and poor oral hygiene; g) known sensitivity to dronabinol, cannabidiol, propylene glycol, ethanol or peppermint oil (used in Sativex buccal spray); h) unstable medical conditions; i) pregnant or breast-feeding; j) currently taking psychotropic medication for any indication other than treatment of insomnia; or k) holding a job that involves driving or operating heavy machinery.

Reasons for terminating study participation included one or more of the following: severe adverse effects; major protocol violations; loss to follow-up; pregnancy; or withdrawal of consent.

### Procedures

Participants were recruited by way of media advertisements and flyers indicating basic study parameters placed within the Greater Toronto Area (Canada). Following a brief telephone screening interview, prospective participants meeting most eligibility criteria were invited for an in-person interview for consent procedures and to assess eligibility (Baseline visit, week 0, see S1 Table). Baseline evaluations included: Structured Clinical Interview for DSM-IV Axis I Disorders, Patient Version (SCID-I/P) [32]; demographic assessments; psychiatric/medical evaluation and physical examination by a study physician (including weight (kg), vital signs (temperature, pulse, blood pressure and respiration rate) and medical history); breath carbon monoxide (CO); 12-lead electrocardiogram (EKG); blood work, including complete blood count (CBC), electrolytes, renal and liver function tests; serum pregnancy test (beta-HCG) (females); female participants were asked if they were lactating; ten-panel urine toxicology screen; Brief Psychiatric Rating Scale (BPRS) [33]; Systematic Assessment for Treatment Emergent Events (SAFTEE) [34]; Hamilton Anxiety Scale (HAM-A) [35]; Hamilton Depression Rating Scale (HDRS) [36]; Timeline Followback (TLFB) for cannabis, tobacco, caffeine and alcohol [37]; Fagerstrom Test for Nicotine Dependence (FTND) [38]; Addiction Severity Index (ASI) [39]; Beck Depression Inventory (BDI) [40]; Drug Effects Questionnaire (DEQ) [41]; Profile of Mood States [42]; Marijuana Withdrawal Checklist (MWC) [43]; Marijuana Craving Questionnaire–Short Form (MCQ-SF) [44]; and the St Mary’s Hospital Sleep Questionnaire (SMHSQ) [45].

Eligible participants were enrolled by the principal investigator in the study and randomized in blocks of 10 to one of the two groups (nabiximols vs. placebo) in a 1:1 ratio and in a double blind manner by the participating pharmacy. All study staff except for the participating pharmacy were blinded after assignment to interventions. Participants started treatment on the first visit following baseline assessment and completed two weekly assessment visits during the 12-week course of treatment. One of the weekly visits corresponded to the MET/CBT visit (approximately 1 h in duration). The other weekly visit included the same assessments as in Baseline (listed above) except for the SCID-I, demographic assessments, medical history and EKG. During the trial, regular ten-panel urine drug tests were performed and blood samples were collected for cannabinoid analyses (S1 Table).

Participants received up to CDN$ 855 in compensation for their participation. To engage participants and maximize returns to CAMH for daily visits, participants drew a ticket for a chance to win a prize. Prizes ranged from tickets containing motivational messages, a pen or notepad, or gift cards from $5 to $50. Additionally, participants were provided transportation fare tokens (or their cash value of $6) to assist with attending study visits.

### Nabiximols dosing

Nabiximols and placebo sprays were donated by GW Pharmaceuticals. New nabiximols vials were provided to participants during the weekly study visits as scheduled. Participants were required to bring their previous vial to each study session. Medication use was assessed by weighing each vial before use, during each study visit and upon return. Used vials were returned to the study hospital’s pharmacy for disposal.

On the first treatment visit, following the baseline visit, participants were instructed in the use of study medication and took their first dose observed by study staff and remained at study site for two hours to ensure tolerability of medication, to assess for any idiopathic adverse events, and to evaluate the safety of study participation. Participants were instructed to self-titrate the study medication (as per the schedule shown in supporting information S2 Table). Maximum doses of nabiximols were reached at day 10 of the treatment course. The target quit date for cannabis was set at day 21. Participants were provided with a ‘smoking diary’ during the first study visit and instructed to enter information regarding the frequency of cannabis and medication use each study day. Cannabis use was recorded for all possible forms or administration routes (joints, pipes, ingested, etc.) [46].

### MET/CBT intervention

All participants received a weekly MET/CBT session with a trained clinical psychologist for 12 weeks. This was an enhancement of a nine-week MET/CBT intervention that was previously studied for the treatment of cannabis dependence and found to be effective [47]. The intervention emphasized the development of motivation for change and the implementation of skills to reduce and abstain from cannabis use, using the Brief Counseling for Marijuana Dependence manual published by the Substance Abuse and Mental Health Services Administration (SAMHSA) [48]. Studies of psychosocial interventions in cannabis dependence showed that more intensive interventions had a more sustainable outcome [47]; hence the MET/CBT sessions continued for the full 12 weeks of treatment. The current SAMHSA manual provides an outline for this intervention for eight weeks and provides four additional elective topics. In this study, participants received the eight weekly sessions as outlined and were then provided all four of these elective topics to maintain consistency and the 12-week intervention length. These elective topics were identical amongst all participants. Inclusion of this intervention allowed us to assess the additive value of treatment with nabiximols for cannabis dependence.

### Therapist competence and treatment adherence

All treatment sessions were audio-recorded and an evaluator (LCQ) blind to treatment assignment reviewed 14 sessions to assess therapist competence and treatment fidelity. The Session Rating Form from the Brief Counseling for Marijuana Dependence: A Manual for Treating Adults [49] was utilized to evaluate therapists’ adherence to manual guidelines. The Cognitive Therapy Scale–Revised (CTS-R; [50]) and Motivational Interviewing Assessment: Supervisory Tools for Enhancing Proficiency (MIA-STEP;[51]) were utilized to evaluate the therapists’ skill levels.

### Cannabinoid concentrations

Urine and blood samples for THC and metabolites analysis were collected throughout the study. Urine samples were taken at the beginning of the visit, whereas blood samples were collected at the completion of the study visit to standardize time since the last spray. Urine creatinine was determined by the CAMH laboratory and cannabinoid concentrations by Dr. Huestis’ Chemistry and Drug Metabolism laboratory at the National Institute on Drug Abuse (NIDA). Participants were considered abstinent based on self-reports from TLFB and smoking diaries. As THC is a component of nabiximols, THC metabolites are naturally expected to be present in the samples for both cannabis-using participants as well as abstinent participants using nabiximols. Thus, standard urine sampling for THC metabolites could not be easily used as a measure of abstinence. To address this, we complemented our analysis with qualitative measures in an attempt to determine exclusive nabiximols use. Urine and plasma samples were analyzed at NIDA using previously described methodology [52,53] (see supporting information for details S1 File).

### Abstinence verification

Verification of abstinence from cannabis or other drugs was based on self-reports (smoking diary and TLFB), preliminary urine drug tests (QuickScreen^TM^ Cup Multi Drug Screening Test, Confirm Biosciences, San Diego, CA, USA) and THC and metabolite concentrations in urine and plasma specimens. The cutoff concentrations for QuickScreen^TM^ Cup Multi Drug Screening test were; Barbiturates at 200 ng/mL, Benzodiazepines at 200 ng/mL, Methadone at 300 ng/mL, Amphetamine at 1000 ng/mL, Methamphetamine at 500 ng/mL, Cocaine metabolite (Benzoylecgonine) at 300 ng/mL, THC metabolite (THCA) at 50 ng/mL, Opiates at 300 ng/mL, Oxycodone at 100 ng/mL and PCP at 25 ng/mL. Daily cannabis (and other drugs) use was self-reported using the TLFB questionnaire and smoking diary. The abstinence rate (seven-day point prevalence and days/week use of cannabis) was measured one week after the end of the medication phase.

### Statistical analysis

The main objective of the study was to assess tolerability and possible trends for efficacy of nabiximols for the treatment of cannabis dependence. We hypothesized that the cannabis use in the treatment group would decrease by at least 50% compared to pre-treatment levels. Power calculation estimated that a sample of 18 subjects per group will have sufficient power to detect a difference in abstinence rates across the two study groups if the proportion of subjects who are abstinent in the study group is 50% or higher (based on Chi-square or logistic regression analysis). All analyses were done on an intention-to-treat basis. Data collected during weekly visits are presented as means ± SD. Data were analyzed using a Generalized Linear Mixed Model (GLMM) with the intervention group treated as a between-subjects factor and time (treatment week) as a within-subjects factor and no covariates. Case intercept random effects were included in the analysis. One-way analysis of variance (ANOVA) was used to determine differences between nabiximols and placebo conditions when appropriate. Differences were considered statistically significant at p < .05. Statistical software IBM SPSS Statistics version 21.0 was used for analysis. Missing data were handled by Maximum Likelihood estimation, which makes use of all available information in the data base.

## Results

### Study recruitment and retention

**Fig 1:**
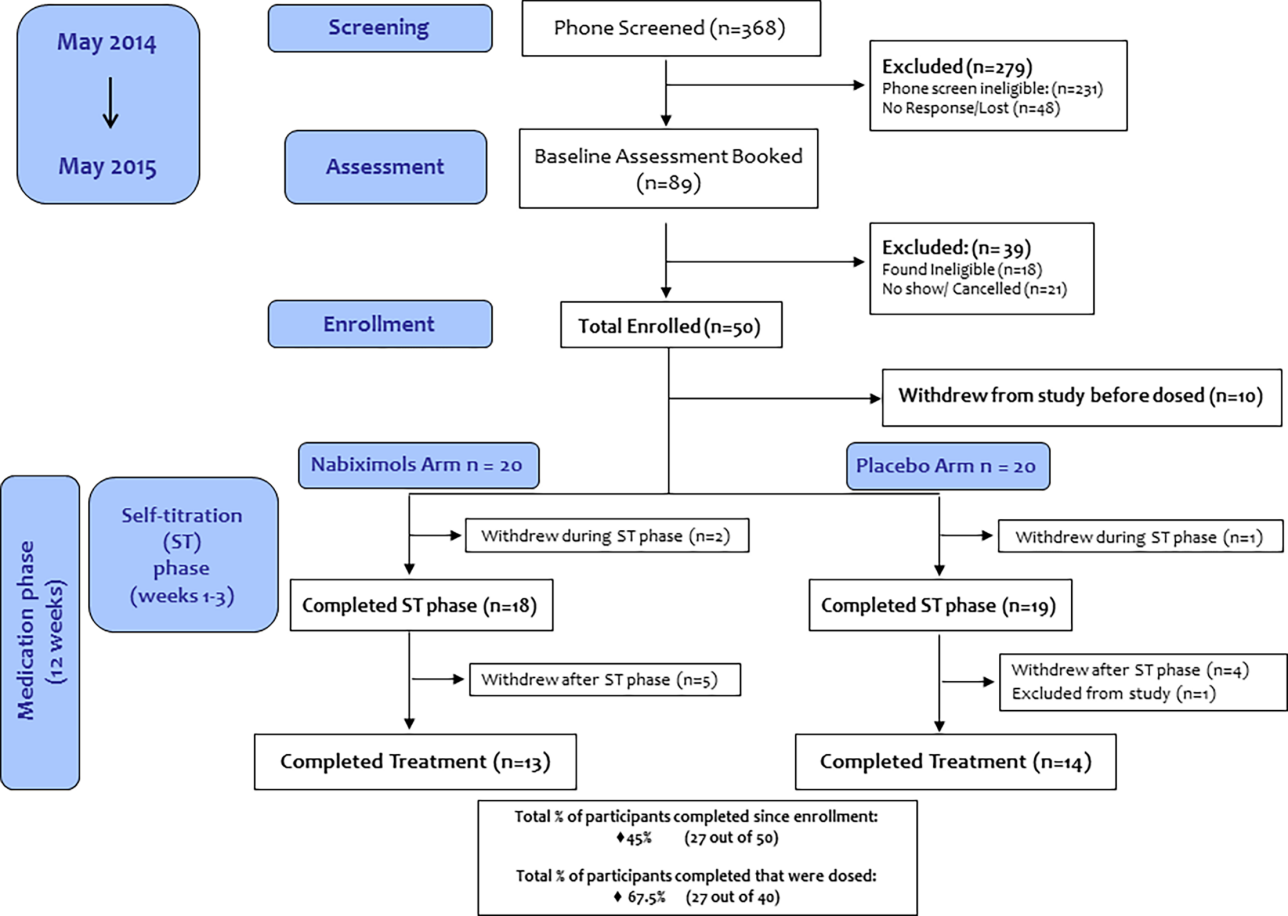
Consort flow diagram. Diagram shows the number of participants at each stage of the study.

The study flow diagram is presented in Fig 1. A total of 89 participants were invited for a screening assessment, between May 2014 and May 2015. Twenty-one participants did not attend the screening visit; 18 were ineligible (8 did not meet criteria for current cannabis dependence, 4 had medical conditions, 3 Axis I disorders, 1 did not consent, 1 had conflicts with schedule requirements and 1 quit cannabis use prior to the study). A total of 50 participants were deemed eligible to receive nabiximols, but only 40 were dosed (10 participants were eligible but no longer interested in study participation, e.g. unable to commit to time requirement for study, reported quitting on his/her own, lost to follow-up, unresponsive with study personnel to schedule first visit and not specified reason). Twenty-seven participants completed the study protocol’s entire experimental sequence; 12 participants withdrew before completing the study (7 from nabiximols group and 5 from placebo group) and 1 participant (from placebo group) was excluded before completing the treatment phase (incompatible schedule with the study).

### Demographics

Table 1 shows demographics, substance use assessments, and psychosocial functioning scores for participants. Average reported use of cannabis for participants at baseline is as follows: 6.4 days/week (SD = 1.3), consuming an average of 6.0 g of cannabis /week (SD = 5.0). Participants assigned to the nabiximols group (n = 20) reported using cannabis an average of 6.7 days /week (SD = 0.8) at baseline, consuming 6.2 g/week (SD = 5.0). Participants in the placebo group (n = 20) reported using cannabis 6 days /week at baseline (SD = 1.8), consuming an average of 5.9 g /week (SD = 5.0).

**Table 1:** Baseline characteristics for treatment groups. Table shows demographics, substance abuse assessments and psychosocial functioning scores. Values represent mean values (SD).

Characteristics	Total Enrolled n = 40	
Treatment group	Placebo (n = 20)	Nabiximols (n = 20)
**Demographics, No. (%)**		
Age, years, mean (SD)	35.3 (13.1)	30.7 (10.4)
Male	14 (70)	15 (75)
Female	6 (30)	5 (25)
White, Non-Hispanic^†^	13 (65)	11 (55)
Mixed^#^	4 (20)	3 (15)
Asian	2 (10)	4 (20)
Black	0 (0)	2 (10)
Latin American	1 (5)	0 (0)
Aboriginal	0 (0)	0 (0)
College Degree/University	11 (55)	14 (70)
Full-time Employed	4 (20)	1 (5)
Married /Common-Law	3 (15)	4 (20)
**Substance Abuse Assessment, mean (SD)**		
Addiction Severity Index		
Employment	0.3 (0.3)	0.3 (0.3)
Medical Status	0.1 (0.2)	0.1 (0.1)
Psychiatric Status	0.3 (0.5)	0.2 (0.4)
Family/Social	0.1 (0.1)	0.1 (0.1)
Alcohol Use	0.1 (0.1)	0.1 (0.1)
Drug Use	0.2 (0.1)	0.2 (0.1)
Legal Status	0.0 (0.0)	0.1 (0.2)
Fagerstrom Test for Nicotine Dependence	0.8 (1.9)	0.6 (1.4)
**Psychosocial functioning scores, mean (SD)**		
Hamilton Anxiety	2.3 (3.0)	3.5 (3.6)
Hamilton Depression Rating Scale	2.4 (4.3)	3.3 (2.8)
Beck Depression Inventory	6.6 (6.0)	7.6 (5.7)
Brief Psychiatric Rating Scale	19.9 (2.9)	21.2 (4.7)
Profile of Mood States	24.4 (38.2)	31.2 (26.7)
St. Mary’s Sleep Questionnaire		
Sleep Latency (min)	34.3 (34.5)	55.5 (48.8)
Sleep Duration (min)	433.6 (140.0)	385.3 (91.0)
Sleep Quality	15.4 (3.7)	16.8 (2.0)

† White (n = 10); White: North American (n = 8);White: European (n = 5);White: European/North American (n = 1)

# Mixed Background (n = 3); White & Hispanic (n = 1) or Middle Eastern (n = 1); Black: Caribbean & White: North American (n = 1); Indian (n = 1)

### Use of medication

**Fig 2:**
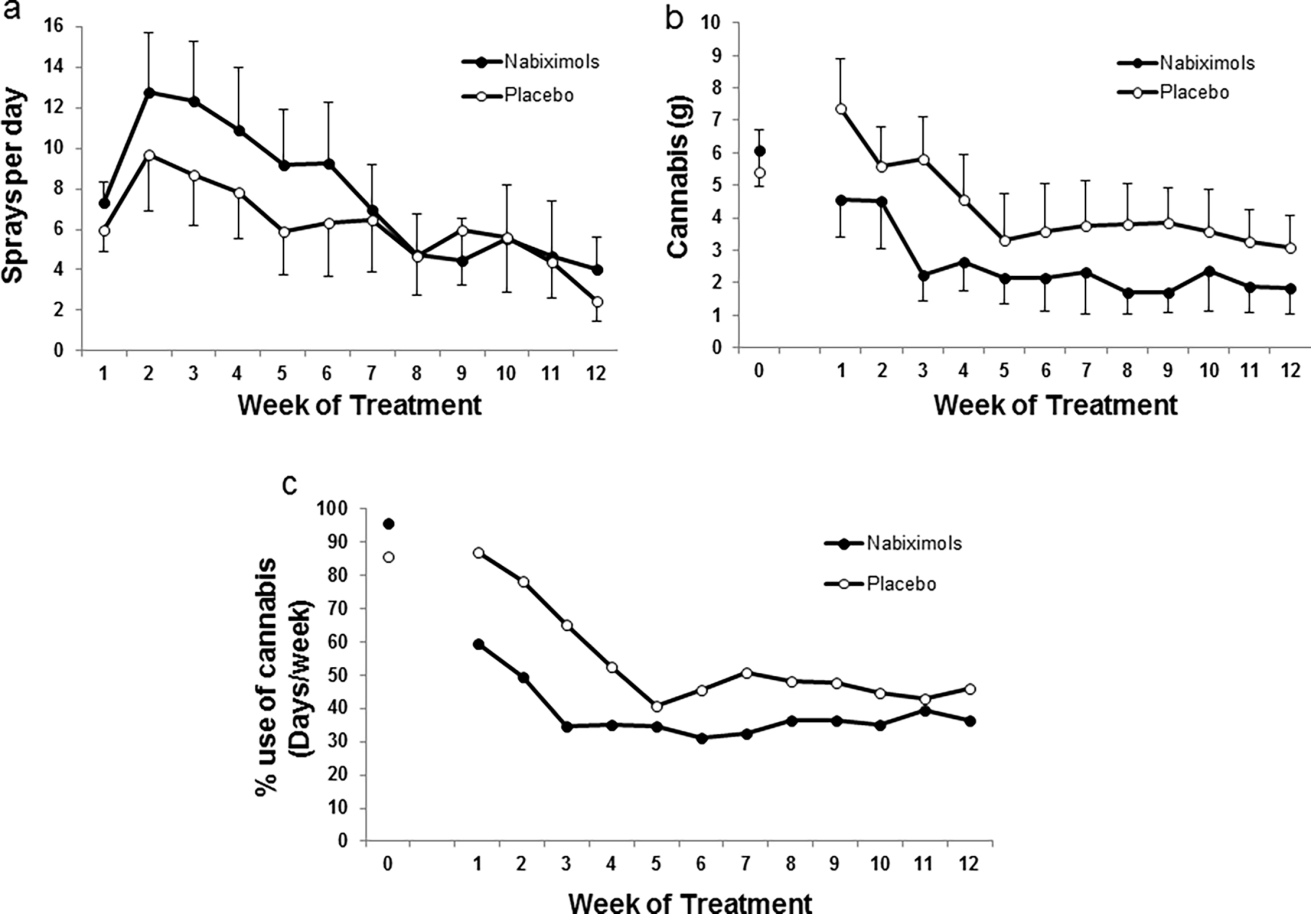
Study medication rates/effects in cannabis use. Circles (white placebo, black nabiximols) represent mean (+SEM). In a) self-titrated medication (sprays/day) as reported in the smoking diary. In b) total average cannabis intake (g) per week as reported in the timeline followback (TLFB) (week 0) and smoking diary (weeks 1–12). In c) mean percentage of days using cannabis (nabiximols n = 20–13, placebo n = 20–14).

**Fig 3:**
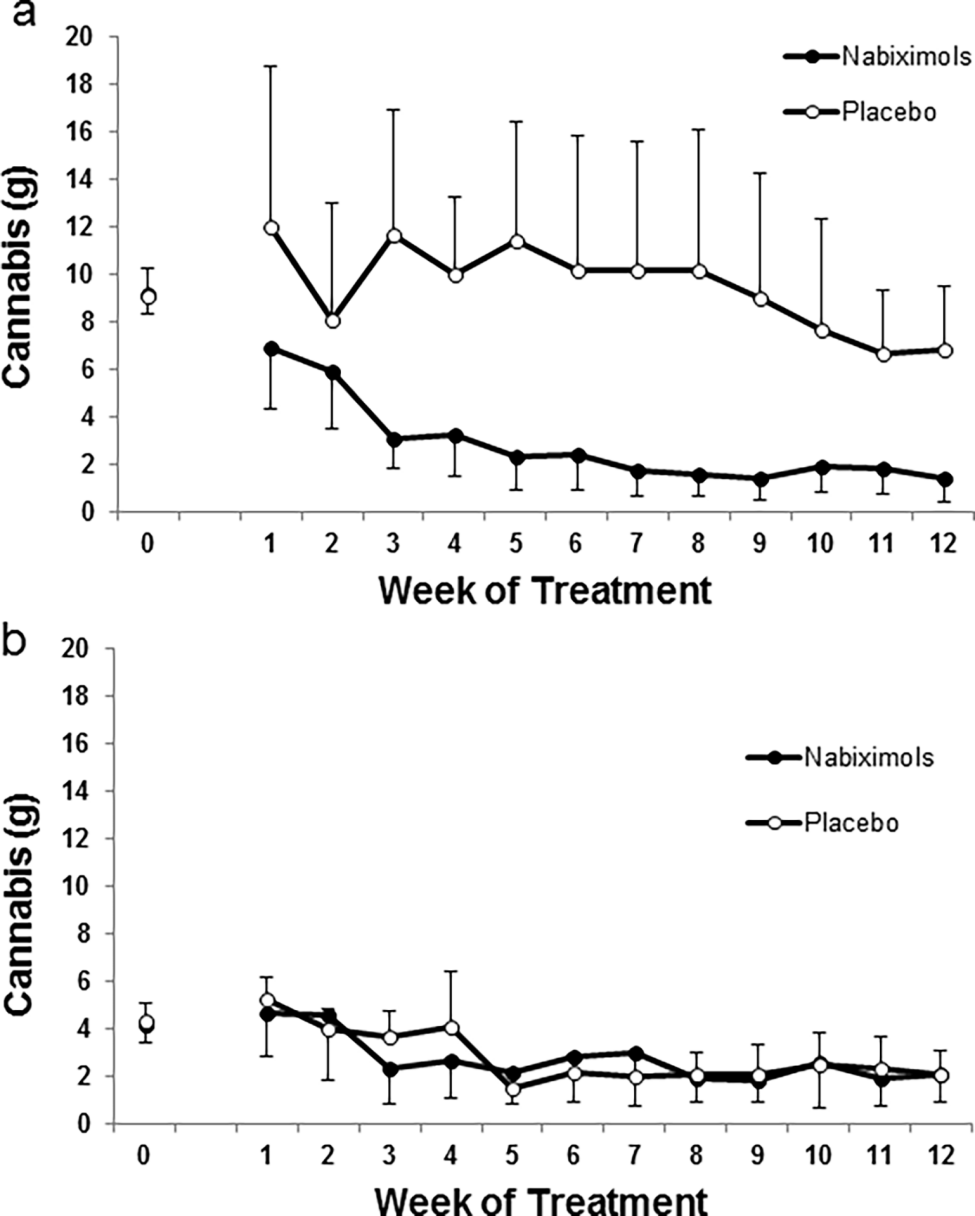
High/low study medication effects in cannabis use. Circles (white placebo, black nabiximols) represent mean (+SEM) for total cannabis intake (g) per week as reported in the timeline followback (TLFB) (week 0) and smoking diary (weeks 1–12). In a) high medication users’ subgroup (≥ 20 sprays on any treatment day) (n = 5 and 3 for nabiximols and placebo, respectively), in b) low medication users sub-group (< 20 sprays at any treatment day) (n = 8 and 11 for nabiximols and placebo, respectively).

Participants’ self-reported use of medication (nabiximols or placebo) is displayed in Fig 2A. A main objective for this study was to assess tolerability of the self-titrated dosage. Medication was well tolerated by all participants and no serious adverse events were observed in any of the experimental conditions. In the nabiximols group, average doses ranged from 4.1 to 12.8 sprays/day (11.0 THC/10.2 mg CBD to 34.5 THC/31.9 mg CBD). In the placebo group, the average number of sprays ranged from 2.5 to 9.7 sprays/day. There was high variability in the number of sprays used by participants. Therefore, we performed an additional analysis of the study outcomes by sub-dividing each treatment group into a high medication user sub-group (≥ 20 sprays on any treatment day) and a low medication user sub-group (< 20 sprays on all treatment days) (see Fig 3 and supporting S3 and S4 Figs).

The subjective effects of medication were evaluated by using the DEQ. Participants were not able to differentiate between nabiximols and placebo effects even at the higher medication rates (i.e. high medication user subgroup, see S5 Fig). GLMM analyses showed a significant effect of time (F_1,160_ = 7.261, p < .001), but no significant differences between treatment conditions (F_1,40_ = 0.585, p = .449) and no significant time x treatment interaction (F_1,160_ = 0.393, p = .813).

### Cannabis use

Cannabis use decreased in both groups (Fig 2A). GLMM analyses showed a significant effect of time (F_12,377.9_ = 7.159, p < .001), but no significant differences in cannabis use between treatment conditions (F_1,36.5_ = 1.876, p = .179) and no significant time x treatment interaction (F_12,377.9_ = 0.787, p = .664). This reduction in cannabis use during the trial was also assessed by analyzing the percentage of days of cannabis use (Fig 2C). GLMM analyses showed a significant effect of time (F_12,382.8_ = 15.166, p < .001), but no significant differences between treatments in the number of days of cannabis use (F_1,36.3_ = 1.114, p = .298) and no significant time x treatment interaction (F_12,382.8_ = 1.292, p = .221). The second main objective for this study was to evaluate abstinence rates in the nabiximols group vs placebo group. No significant difference was observed in abstinence rates between the two groups. The seven-day point prevalence cannabis abstinence after the medication phase was 30.8% (n = 4) for nabiximols and 42.9% (n = 6) for placebo, respectively. One of the 13 participants completing the study in the nabiximols group quit cannabis on the target day (day 21) and remained abstinent for the rest of the study. The remaining participants in the nabiximols group (n = 12) reduced their cannabis use and 5 remained abstinent for at least 4 consecutive weeks (range 4–18 weeks). One of the 14 participants in the placebo group quit cannabis before being dosed and remained abstinent during the study. The remaining participants in the placebo group (n = 13) reduced their cannabis use and 6 remained abstinent for at least 4 consecutive weeks (range 4–21 weeks). Cannabis use decreased a 70.5% (from 6.1 to 1.8 grams) in the nabiximols group at end of treatment vs a 42.6% (from 5.4 to 3.1 grams) reduction of cannabis use in the placebo group. Different levels of cannabis use were observed in the high vs low medication use sub-groups. A trend for reduction of cannabis use was observed in high nabiximols users vs placebo, whereas the cannabis use was similar in the nabiximols and placebo groups in the low medication use sub-groups (Fig 3A and 3B, respectively). GLMM analyses for grams of cannabis consumed in the high nabiximols user’s group showed a significant effect of time (F_12,96_ = 3.635, p < .001), no significant differences between treatments (F_1,8_ = 3.499, p = .098) and a significant time x treatment interaction (F_12,96_ = 2.480, p < .01). A subsequent one-way ANOVA showed no significant differences between treatments at any time point during treatment.

### Abstinence verification and other drug use

Cannabis abstinence verification was based on self-reports (smoking diary and TLFB). Cannabis and other drug use was confirmed in urine drug tests and by analyzing THC and THC metabolite concentrations in the urine and plasma specimens collected. Daily cannabis (and other drugs) use was self-reported using the TLFB questionnaire (S2 Fig) and the smoking diary (Fig 2B). Results of the TLFB for cannabis were consistent with participants’ smoking diaries. Additionally, the TLFB showed no significant compensatory increases in use of other substances when participants reduced their cannabis use or remained abstinent (S2 Fig).

Participants self-reported compliance in the use of nabiximols by above-described recording methods (Fig 2A). Self-reports of medication use matched the vials’ weight assessments (see Fig 2A and S6 Fig). Results were further supported by the analysis of plasma and urinary cannabinoids, which are consistent with the adherence of participants to the experimental conditions (see Plasma and Urinary Cannabinoids section below).

Use of other illegal drugs was self-reported (e.g., in the smoking diary) and confirmed by means of the urine tests performed on site. Five participants in the placebo group and four participants in the nabiximols group had other illegal drug use during the study. More specifically, participants in the placebo group totaled 10 visits with results positive for other illegal drugs (6 for cocaine, 3 for opioids, 1 for amphetamine). Participants in the nabiximols group totaled 4 visits positive for other illegal drugs (2 for cocaine, 2 for amphetamine).

### Effects of nabiximols on cannabis withdrawal

**Fig 4:**
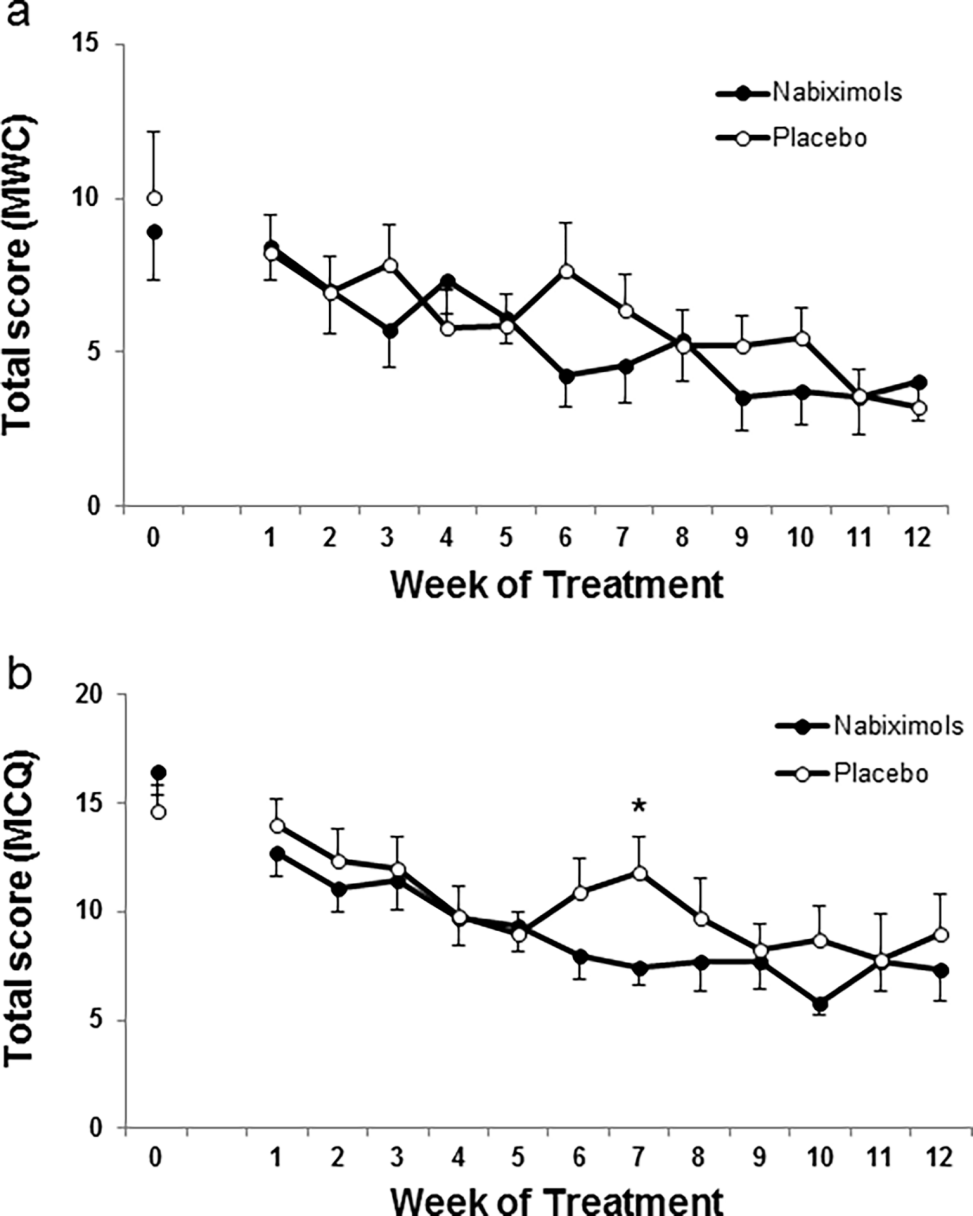
Cannabis craving and withdrawal. Circles (white placebo, black nabiximols) represent mean (+SEM). In a) cannabis withdrawal from the Cannabis Withdrawal Checklist (CWC). In b) craving for cannabis from the Marijuana Craving Questionnaire (MCQ). Generalized Linear Mixed Model (GLMM) analyses followed by one-way ANOVA, * (p < .05) vs nabiximols group.

Cannabis withdrawal was assessed by the MWC. Total scores for MWC progressively decreased along the 12-week treatment in both groups (Fig 4A). GLMM analyses showed a significant effect of time (F_12,349.6_ = 6.207, p < .001), but no significant differences between treatments in withdrawal scores (F_1,41.0_ = 0.290, p = .593) and no significant time x treatment interaction (F_12,349.0_ = 0.848, p = .601). As reported above for cannabis use, dissimilar patterns for withdrawal scores were observed in the high/low medication sub-groups (supporting information S3 Fig); however, differences between nabiximols and placebo groups were not statistically significant.

### Effects of nabiximols on cannabis craving

Total craving scores progressively decreased along the 12-week treatment in both groups (Fig 4B). GLMM analyses showed a significant effect of time (F_12,344.4_ = 17.932, p < .001), no significant differences between treatments in craving scores (F_1,40.3_ = 0.615, p = .438), but a significant time x treatment interaction (F_12,344.4_ = 1.938, p < .05).

Total craving scores decreased along the 12-week treatment in both the high and low medication sub-groups (supporting information S4A and S4B Fig, respectively). GLMM analyses in the high medication sub-group showed a significant effect of time (F_12,90.1_ = 10.386, p < .001), no effects of treatment (F_1,8.1_ = 1.200, p = .305) but a significant time x treatment interaction (F_12,90.1_ = 2.741, p < .01). GLMM analyses showed no significant differences between treatments in craving scores in the low medication sub-group.

### Urinary and plasma cannabinoids

Creatinine-normalized urine cannabinoid concentrations (ng/mg) for nabiximols and placebo groups are shown in Table 2. Urinalysis showed significantly higher concentrations of CBD in the nabiximols group as compared to placebo. GLMM analyses showed significant effects of time (F_3,69.6_ = 3.699, p < .05) and treatment (F_1,24.6_ = 6.573, p < .05) and a significant time x treatment interaction (F_3,69.6_ = 3.699, p < .05). Concentrations of CBD went up to 28 ng/mg at week 8 of treatment with nabiximols but were barely above the limit of quantification in the placebo group. On the other hand, no significant effects of time or treatment with nabiximols or placebo were observed on THC, 11-hydroxy-THC (11-OH-THC), 11-nor-9-carboxy-THC (THCCOOH) and cannabinol (CBN) concentrations.

**Table 2:** Cannabinoids of interest in urine were quantified using two-dimensional gas chromatography-mass spectrometry (2D-GCMS). Table represents creatinine-normalized mean urine Δ9-tetrahydrocannabinol (THC), 11-hydroxy-THC (11-OH-THC), 11-nor-9-carboxy-THC (THCCOOH), cannabidiol (CBD) and cannabinol (CBN) concentrations for nabiximols and placebo groups.

**Cannabinoids of interest in urine**								
	**Nabiximols**	**Week of Treatment**						
		**0**		**4**		**8**		**12**
**Analytes(ng/mg)**	**THC**	1		2.8		2		1.3
	**11-OH-HC**	6.9		17.5		13.6		11
	**THCCOOH**	398		577		542		283
	**CBD**	0.2		24.5		27.9		5.3
	**CBN**	0.4		0.2		0.4		0.3
	**Placebo**	**Week of Treatment**						
		**0**		**4**		**8**		**12**
**Analytes(ng/mg)**	**THC**	1.7		0.4		1.4		1.7
	**11-OH-HC**	15.3		7.5		8.5		11
	**THCCOOH**	507		282		307		492
	**CBD**	0		0		0		0
	**CBN**	0		0.1		0		0.1
**Cannabinoids of Interest in Urine**								
	**Nabiximols**	**Week of Treatment**						
		**0**		**4**		**8**		**12**
**Analytes(ng/mg)**	**THC**	1		2.8		2		1.3
	**11-OH-HC**	6.9		17.5		13.6		11
	**THCCOOH**	398		577		542		283
	**CBD**	0.2		24.5		27.9		5.3
	**CBN**	0.4		0.2		0.4		0.3
	**Placebo**	**Week of Treatment**						
		**0**		**4**		**8**		**12**
**Analytes(ng/mg)**	**THC**	1.7		0.4		1.4		1.7
	**11-OH-HC**	15.3		7.5		8.5		11
	**THCCOOH**	507		282		307		492
	**CBD**	0		0		0		0
	**CBN**	0		0.1		0		0.1

Plasma THC, 11-OH-THC, THCCOOH, THC-glucuronide (THC-glu), THCCOOH-glucuronide (THCCOOH-glu), CBD and CBN concentrations did not significantly differ between groups (see supporting information S3 Table).

### Effects of nabiximols on physiological measures

Nabiximols and placebo treatment produced no changes in body weight, blood pressure, respiration rate or body temperature during treatment. Mean CO concentrations before treatments were 7.9 ppm (SD = 8.2) for placebo and 7.8 ppm (SD = 5.5) for nabiximols group. At the end of treatment mean CO concentrations were 10.2 ppm (SD = 14.4) for placebo and 4.7 ppm (SD = 2.4) for nabiximols group. Statistical analysis showed no significant effects of time (F_12,337.6_ = 1.004, p = .445), treatment (F_1,35.7_ = 0.163, p = .689), or significant interaction time x treatment (F_12,337.6_ = 0.643, p = .805) in CO concentrations. Self-reported number of cigarettes used during treatment showed a trend for reduced use of tobacco only in the nabiximols group (S2B Fig), whereas the number of cigarettes in the placebo group remained similar before (week 0) and at the end of treatment (week 12). GLMM analyses for nicotine showed no significant effect of time (F_12,346.5_ = 0.818, p = .632) or treatment (F_1,40.2_ = 0.482, p = .492), but a significant time x treatment interaction (F_12,346.5_ = 2.573, p < .01). Subsequent one-way ANOVA analysis showed no significant differences between treatments at any time point during treatment.

### Adverse events

We did not observe serious adverse events (SAEs) associated with the study medication (nabiximols/placebo). Adverse events observed in the study included some events not related to the study (e.g., mild cold, tension headache or hot flashes) and some expected side effects, such as sleep problems, headaches, or diarrhea. One-way ANOVA showed no significant condition effect in the appearance of the adverse events (F_1,39_ = 0.205, p = .654). Similarly, no treatment effects on depression or anxiety were revealed (p > .05). Sleep latency, duration and quality were evaluated using the SMHSQ. GLMM analyses for sleep latency showed no treatment effects (F_1,37.7_ = 0.003, p = .959) but a significant time effect (F_12,343.7_ = 2.173, p < .05) and time x treatment interaction (F_12, 343.7_ = 2.367, p < .01). Subsequent one-way ANOVA showed no significant differences between treatments at any time point during treatment. GLMM analyses for sleep duration revealed significant treatment effects (F_1,39.2_ = 10.760, p < .01) but no effects of time (F_12,349.5_ = 1.007, p = .441) or time x treatment interaction (F_12, 349.5_ = 0.368, p = .974). No effect in sleep quality was observed.

### MET/CBT

Compliance with MET/CBT treatment was similar between participants in the nabiximols and placebo groups. The nabiximols group totaled 150 visits attended and 36 visits missed during the 12-week treatment. The placebo group members attended 154 visits and missed 43 visits during the overall 12-week treatment course. Therapists closely adhered to the treatment manual. For example, ratings of CBT interventions were higher for sessions focused upon cognitive and behavioral skills building compared to those focused on enhancing motivation or case management. Therapists were above established thresholds of acceptable competence in both the delivery of CBT (CTR-S M = 53.34, SD = 4.47) and MET (MIA-STEP M = 51.78, SD = 8.05).

## Discussion

We observed good tolerability of self-titrated nabiximols dosages in treatment seeking adults with cannabis dependence. No serious adverse events (SAEs) were observed and the rate of adverse events did not differ between treatment arms. Moreover, participants were not able to differentiate between subjective effects associated with nabiximols or placebo treatments. We did not observe significant changes in abstinence rates at the end of treatment in this trial. On the other hand, a higher reduction in the use of cannabis at end of treatment was observed in the nabiximols group. Additionally, sub-analyses showed a trend for stronger reduction of cannabis use in participants using high doses of nabiximols. Nabiximols was able to reduce cannabis craving despite the greater reduction of cannabis use in this group as compared to placebo (71% vs 43%). In contrast, no effects on cannabis withdrawal scores were observed in this study.

We were expecting that this potentially high medication dosage (up to 113.4 of THC/105 mg of CBD) might be well-tolerated since the participants already had developed tolerance due to their cannabis use. Moreover, in previous studies, we observed that high fixed dosages of nabiximols (108 mg THC and 100 mg CBD, equivalent to 40 sprays) were well-tolerated in non-treatment-seeking cannabis dependent adults [30]. In an initial open label phase for this study, self-titrated nabiximols was 28.7 sprays/day (equivalent to 77.5 mg THC / 71.7 mg CBD) [54], which was similar to the 29.7 sprays/day average intake observed in non-treatment seeking individuals during cannabis abstinence [30]. However, mean intake for the present study was only 8.1 (SD = 3.2) sprays/day for the nabiximols group (equivalent to 21.9 mg THC / 20.3 mg CBD) and 6.6 (SD = 1.7) sprays/day for the placebo group, which seem much lower than in the above studies. Urinary concentrations of CBD were compatible with compliance with the study medication in the nabiximols group.

Overall, participants in the study reduced their cannabis use as compared to baseline. We did not observe significant changes in abstinence rates at the end of treatment in this trial. However, the decrease in cannabis use at the end of treatment in the nabiximols group exceeded the 50% proposed as the main hypothesis for this study, while the reduction in the placebo group did not reach this benchmark. Similarly, other studies also showed a significant reduction in the use of cannabis following nabiximols treatment [31,54]. On the other hand, we did not find significant differences in the use of cannabis during the course of treatment between treatment groups. The above commented scarce use of medication and reduced sample size in this study might account for the above results. Previous studies using a short-duration treatment with nabiximols also reported no differences in use of cannabis [31].

The results in the present study indicated a tendency for better outcomes in nabiximols vs placebo when the study medication was taken in larger amounts; however, the reduced sample size for these subgroups was not sufficient for an effective analysis. In this sense, it might be useful to establish a minimum dose in future studies in larger sample sizes. As expected from previous studies using nabiximols, in this pilot study participants were not able to differentiate between the active medication and placebo treatment, suggesting that the intoxication or the subjective feelings of being high from nabiximols were not perceived as significantly different from placebo [30,31]. In fact, it was previously reported that low nabiximols doses (≤ 16 mg THC, ≤ 15 mg CBD) did not produce clinically significant increases in “good drug effects” [55]. Recent studies suggest that oral CBD, even at high doses (e.g. 800 mg) does not display signals of abuse liability [56]. However, the possibility that oral CBD might modulate the subjective reinforcing effects of THC remains controversial. Studies showed that oral CBD might attenuate the psychotropic effects of oral THC [57]. On the other hand, no effects of oral CBD in ameliorating the reinforcing effects of smoked cannabis were observed [58].

We did not observe compensatory changes in use of caffeine, alcohol or other illegal drugs. However, nabiximols seemed to attenuate tobacco use during the trial, in line with recent studies which suggest CBD as a potential treatment for nicotine dependence [59], though evidence in this respect is still limited [28].

The combination nabiximols + MET/CBT prevented increases in cannabis withdrawal when participants reduced cannabis use. This finding is in agreement with previous laboratory studies using synthetic THC [20,22,24,25] and with recent studies showing stable or reduced cannabis withdrawal following nabiximols [30,31,54]. The literature on CBD effects on cannabis-related addictive behaviors is still scarce but a recent case report suggested that CBD alone might help to cope with cannabis withdrawal [60]. On the other hand, we observed a significant decrease in cannabis craving during the course of treatment. Craving, which is also part of the cannabis withdrawal symptomatology, is the most highly endorsed symptom causing relapse in non-treatment-seeking adults [61,62] and was used frequently in clinical trials [63] and laboratory studies [20]. Indeed, cannabis craving might be linked with cannabis use and it could be used to predict abstinence and cannabis use-related problems [64]. Nevertheless, the validity of cannabis withdrawal and craving measures in predicting the efficacy of therapeutic interventions in subsequent randomized clinical trials is still unclear [65], and the clinical significance of cannabis withdrawal and craving is still being debated [66].

The major limitation for this study is the small sample size. However, it was sufficient to evidence changes in cannabis use as compared to pre-treatment levels, as proposed in the main hypothesis for this study. Another limitation results from the differences in medication intake between participants in the study, further reducing the sample size of these subgroups during analysis. Future studies might establish a minimum dose based on tolerability and safety. An additional limitation is the high abstinence rate in the placebo group (> 40%). This suggests that the behavioral platform was robust and might have diluted the medication effect. Since our study did not include experimental conditions without MET/CBT or without medication we cannot be certain of the effects of nabiximols alone and the contribution of MET/CBT to the observed results. The sample consisted of mostly white males, which results in a limitation in the generalizability of our data. Our study did not include experimental conditions containing THC alone and CBD alone for comparison. Therefore, we cannot be certain of the respective contribution of THC and CBD in the effects observed in this trial. Information regarding the use/effects of cannabis and medication was based mostly on self-reports. However, objective measurements (e.g. vial weight changes and urinary CBD) closely corresponded to the participants’ self-reports for nabiximols usage.

## Conclusions

In summary, our results indicate that the combination nabiximols + MET/CBT was well tolerated. Our observations seem to support the idea that nabiximols may help to decrease cannabis use, with no increase in craving or withdrawal. Our results further suggest that the combination of doses above 20 sprays per day of nabiximols + MET/CBT should be explored further for its potential as a novel treatment approach for CUD.

## Supporting information

S1 Fig Study design. (TIF)

S2 Fig Timeline followback (TLFB) for alcohol, tobacco and caffeine. Circles (in white placebo, in black nabiximols) represent average values (+SEM) of a) Alcohol (standard drinks), b) Tobacco (number of cigarettes) and c) Caffeine (number of soft drinks) during baseline (week 0) and weeks 1–12 of treatment.
(TIF)

S3 Fig High/low study medication effects in cannabis withdrawal. Circles (in white placebo, in black nabiximols) represent average values (+SEM) for cannabis withdrawal as measured using the Cannabis Withdrawal Checklist (CWC). In a) high medication users subgroup (≥ 20 sprays at any treatment day) (n = 5 and 3 for nabiximols and placebo, respectively), in b) low medication users sub-group (< 20 sprays at any treatment day) (n = 8 and 11 for nabiximols and placebo, respectively).
(TIF)

S4 Fig High/low study medication effects in cannabis craving. Circles (in white placebo, in black nabiximols) represent average values (+SEM) for craving scores as determined using the Marijuana Craving Questionnaire (MCQ). In a) high medication users subgroup (≥ 20 sprays at any treatment day) (n = 5 and 3 for nabiximols and placebo, respectively), in b) low medication users sub-group (< 20 sprays at any treatment day) (n = 8 and 11 for nabiximols and placebo, respectively). * (p < .05), ** (p < .01) vs baseline nabiximols group. + (p < .05), ++ (p < .01) vs baseline placebo group. Generalized Linear Mixed Model (GLMM) analyses followed by one-way ANOVA, * (p < .05) vs nabiximols group.
(TIF)

S5 Fig Scores in the drug effects questionnaire (DEQ) during the supervised intake of the study medication. Participants were instructed in the use of study medication and took their first dose observed by study staff and remained at study site for two hours, DEQ measures were determined 30 min (Test #1), 60 min (Test #2), 90 min (Test #3) and 120 min (Test #4) after they took their first dose. Bars (in white placebo, in black nabiximols) represent average (+SEM) values (in mm) for the scores obtained using DEQ visual analog scale. In a) scores for nabiximols and placebo groups (n = 20), in b) high medication users sub-group (< 20 sprays at any treatment day) (n = 5 and 3 for nabiximols and placebo, respectively).
(TIF)

S6 Fig Self-titrated medication as per vials’ weight assessments. Participants were instructed to bring the study medication vials each visit for weight assessments. Weight for each vial was determined before giving it to the participants, during their use and once they were returned to study staff. Circles (white placebo n = 20–14, black nabiximols n = 20–13) represent mean (+SEM) self-titrated medication (sprays/day) for each week of treatment as estimated from vials’ weight (1 spray = 0.1 g).
(TIF)

S1 Table Summary of study assessments. (DOCX)

S2 Table Study medication schedule and maximal number of sprays allowed by study day. The maximum dose of nabiximols for the first two days of treatment was five sprays per day. From the 3rd day, the dose of nabiximols was increased in five sprays per day until it reached a maximum number of 42 sprays per day by week 2 (Day 10). Day 21 was set as the target quit day for cannabis (or before if participants were willing and able to). On week 12 maximum dose of nabiximols allowed was 21 sprays.
(DOC)

S3 Table Cannabinoids of interest in plasma were quantified using liquid chromatography–tandem mass spectrometry (LC-MS/MS) method. Table represents concentrations in plasma specimens for Δ9-tetrahydrocannabinol (THC), 11-hydroxy-THC (11-OH-THC), 11-nor-9-carboxy-THC (THCCOOH), cannabidiol (CBD) and cannabinol (CBN), THC-glucuronide (THC-glu), THCCOOH-glucuronide (THCCOOH-glu) for nabiximols and placebo groups.
(DOCX)

S1 File Supporting information and study protocol. (DOC)

S1 Dataset CONSORT checklist and study Dataset. (ZIP)
