# Supplementary figures and images for: Nabiximols combined with motivational enhancement/cognitive behavioral therapy for the treatment of cannabis dependence: A pilot randomized clinical trial
Source: PLoS One. 2018 Jan 31;13(1):e0190768. doi: 10.1371/journal.pone.0190768 (PMC5791962; doi:10.1371/journal.pone.0190768)

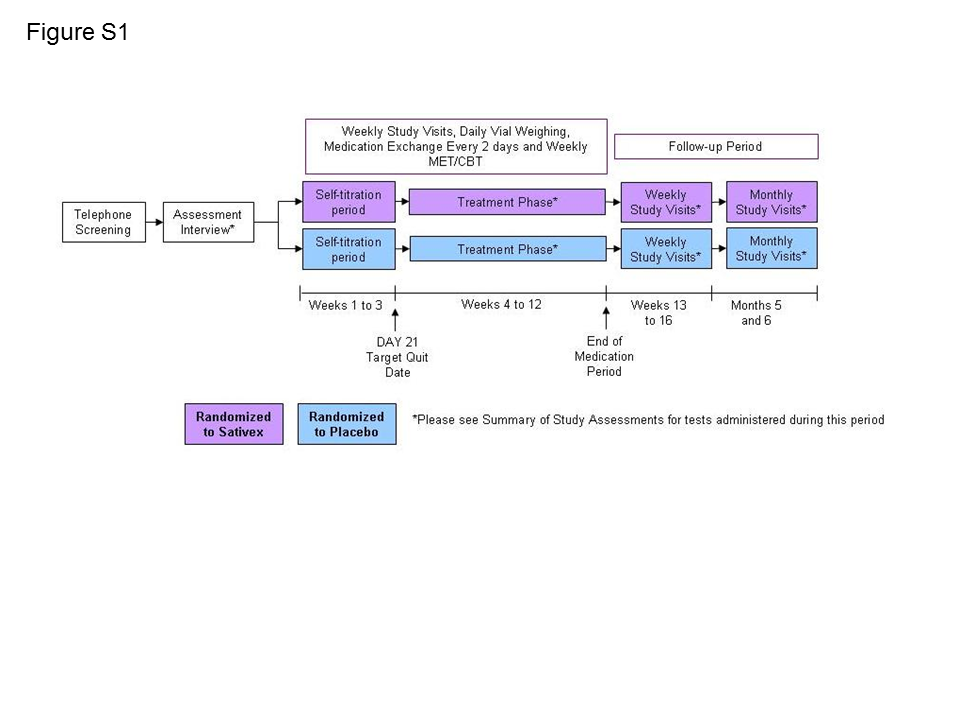

Supplement: S1 Fig — (TIF) [file pone.0190768.s001.tif]

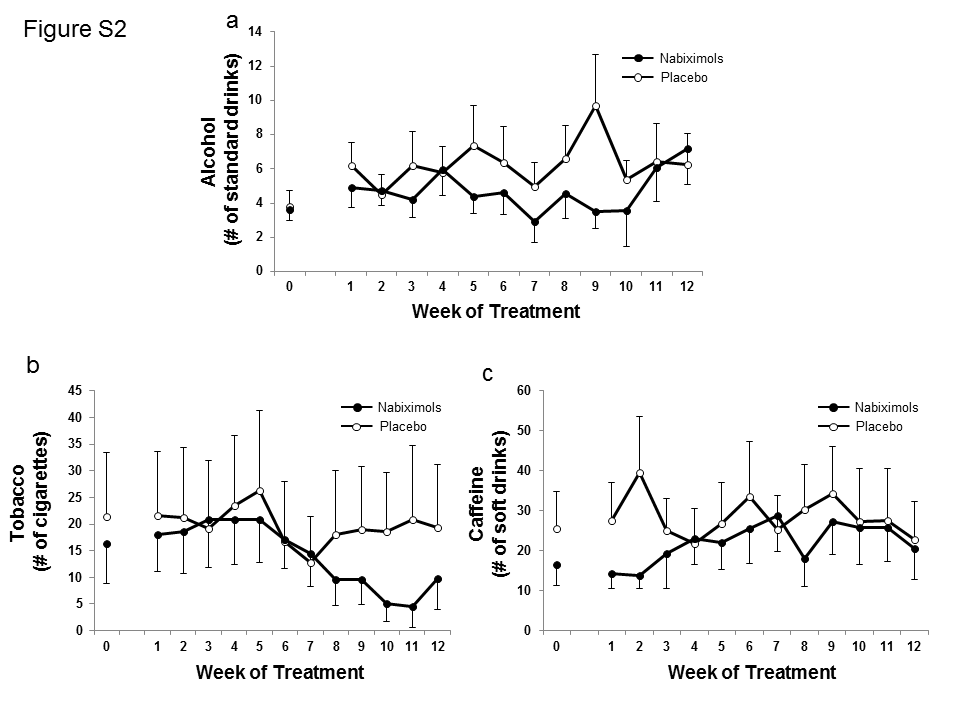

Supplement: S2 Fig — Circles (in white placebo, in black nabiximols) represent average values (+SEM) of a) Alcohol (standard drinks), b) Tobacco (number of cigarettes) and c) Caffeine (number of soft drinks) during baseline (week 0) and weeks 1–12 of treatment. (TIF) [file pone.0190768.s002.tif]

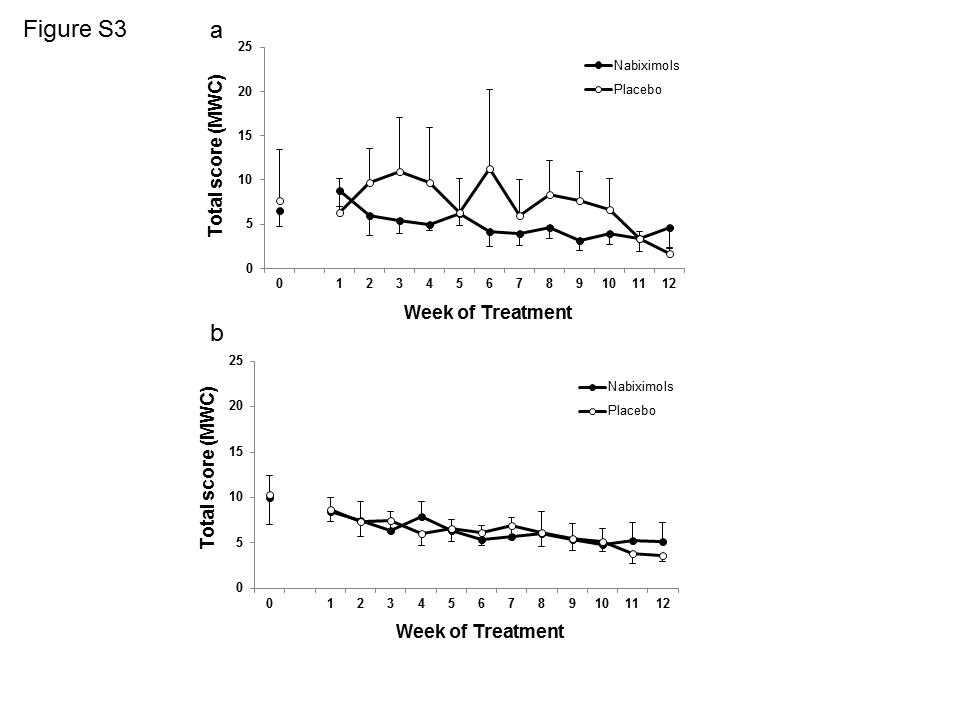

Supplement: S3 Fig — Circles (in white placebo, in black nabiximols) represent average values (+SEM) for cannabis withdrawal as measured using the Cannabis Withdrawal Checklist (CWC). In a) high medication users subgroup (≥ 20 sprays at any treatment day) (n = 5 and 3 for nabiximols and placebo, respectively), in b) low medication users sub-group (< 20 sprays at any treatment day) (n = 8 and 11 for nabiximols and placebo, respectively). (TIF) [file pone.0190768.s003.tif]

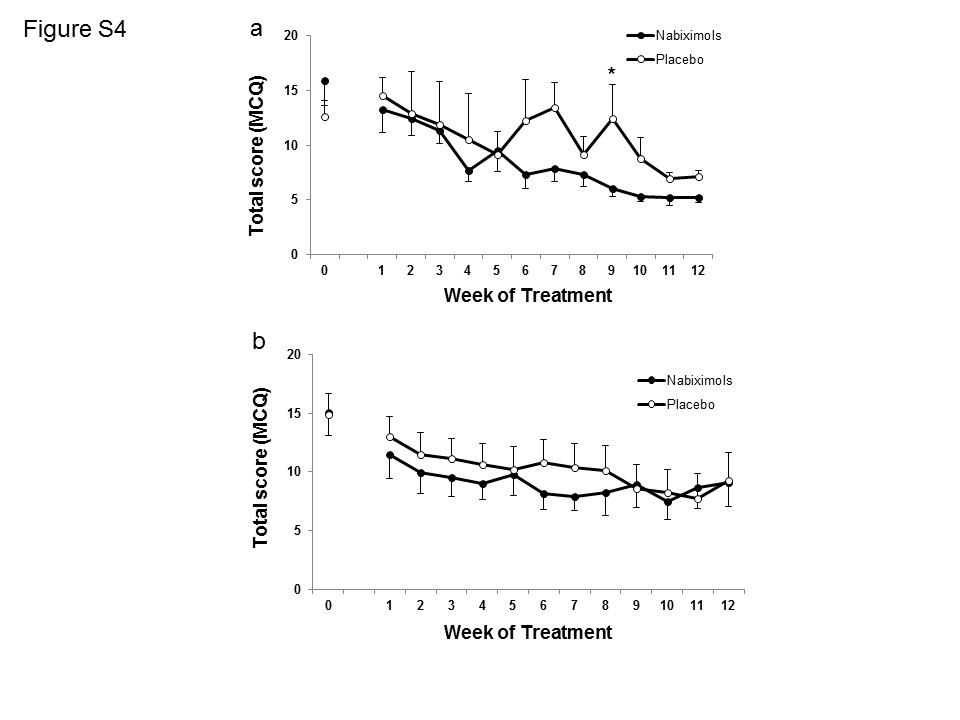

Supplement: S4 Fig — Circles (in white placebo, in black nabiximols) represent average values (+SEM) for craving scores as determined using the Marijuana Craving Questionnaire (MCQ). In a) high medication users subgroup (≥ 20 sprays at any treatment day) (n = 5 and 3 for nabiximols and placebo, respectively), in b) low medication users sub-group (< 20 sprays at any treatment day) (n = 8 and 11 for nabiximols and placebo, respectively). * (p < .05), ** (p < .01) vs baseline nabiximols group. + (p < .05), ++ (p < .01) vs baseline placebo group. Generalized Linear Mixed Model (GLMM) analyses followed by one-way ANOVA, * (p < .05) vs nabiximols group. (TIF) [file pone.0190768.s004.tif]

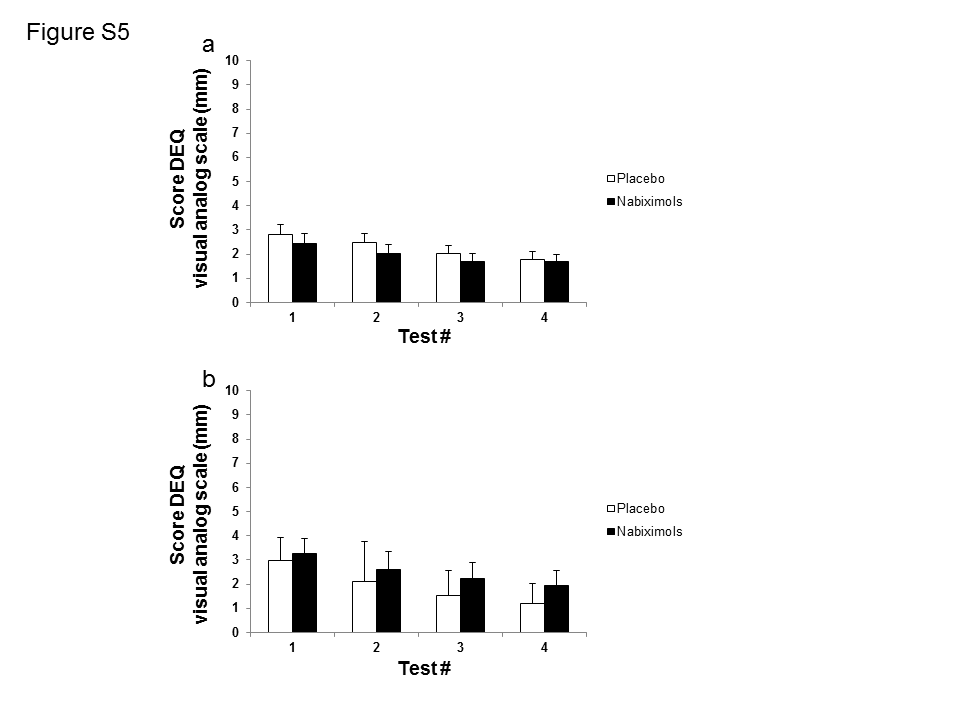

Supplement: S5 Fig — Participants were instructed in the use of study medication and took their first dose observed by study staff and remained at study site for two hours, DEQ measures were determined 30 min (Test #1), 60 min (Test #2), 90 min (Test #3) and 120 min (Test #4) after they took their first dose. Bars (in white placebo, in black nabiximols) represent average (+SEM) values (in mm) for the scores obtained using DEQ visual analog scale. In a) scores for nabiximols and placebo groups (n = 20), in b) high medication users sub-group (< 20 sprays at any treatment day) (n = 5 and 3 for nabiximols and placebo, respectively). (TIF) [file pone.0190768.s005.tif]

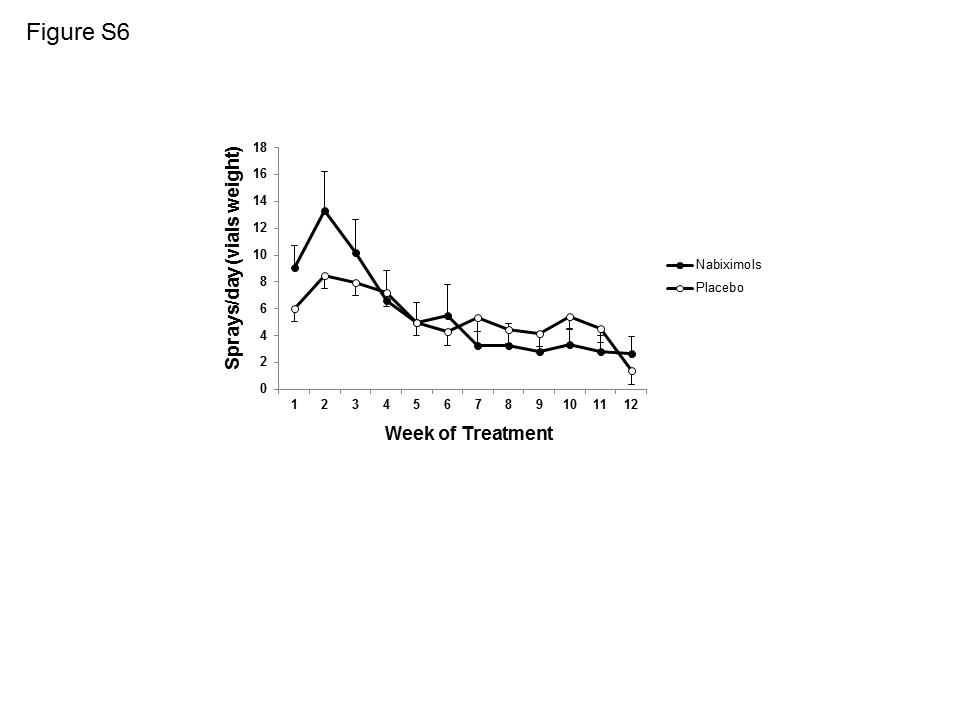

Supplement: S6 Fig — Participants were instructed to bring the study medication vials each visit for weight assessments. Weight for each vial was determined before giving it to the participants, during their use and once they were returned to study staff. Circles (white placebo n = 20–14, black nabiximols n = 20–13) represent mean (+SEM) self-titrated medication (sprays/day) for each week of treatment as estimated from vials’ weight (1 spray = 0.1 g). (TIF) [file pone.0190768.s006.tif]
