# Supplementary material for: Nabiximols combined with motivational enhancement/cognitive behavioral therapy for the treatment of cannabis dependence: A pilot randomized clinical trial
Source: PLoS One. 2018 Jan 31;13(1):e0190768. doi: 10.1371/journal.pone.0190768 (PMC5791962; doi:10.1371/journal.pone.0190768)
Supplement: S1 Table — (DOCX) [file pone.0190768.s007.docx]

**S1 Table**

**Summary of Study Assessments**

| **Assessment** | **Baseline** | **Weekly**  **(Weeks 1-12)**  **medication phase** | **Weekly**  **(Weeks 13-16)** | **Monthly**  **(months 5-6)** |
| --- | --- | --- | --- | --- |
| Psychiatric Evaluation (including SCID) | X |  |  |  |
| Demographic | X |  |  |  |
| Bloodwork (CBC, SMA-12), Urinalysis, EKG | X |  |  | X (end of trial) |
| Physical Exam | X |  |  | X (end of trial) |
| Urine Toxicology | X | X | X | X |
| Vital Signs, Carbon Monoxide | X | X | X | X |
| Serum Pregnancy Test (Females only) | X | If needed | If needed | If needed |
| Urine Pregnancy Test (Females only) | X | X | X | X |
| FTND, Cigarette, alcohol and caffeine Time-line Follow-Back (TLFB) | X | X | X | X |
| Cannabis TLFB, Marijuana withdrawal checklist, MCQ | X | X | X | X |
| BDI, HAM-A, HAM-D, BPRS, Profile of Mood questionnaire, DEQ, SMHSQ | X | X | X | X |
| SAFTEE | X | X |  |  |
| ASI | X |  | One week after medication phase | X (end of trial) |
| Blood for THC metabolite analysis | X | X | X | X |
| Urine for THC metabolite analysis | X | X (two times per week) | X | X |
