# Supplementary material for: Nabiximols combined with motivational enhancement/cognitive behavioral therapy for the treatment of cannabis dependence: A pilot randomized clinical trial
Source: PLoS One. 2018 Jan 31;13(1):e0190768. doi: 10.1371/journal.pone.0190768 (PMC5791962; doi:10.1371/journal.pone.0190768)
Supplement: S2 Table — The maximum dose of nabiximols for the first two days of treatment was five sprays per day. From the 3rd day, the dose of nabiximols was increased in five sprays per day until it reached a maximum number of 42 sprays per day by week 2 (Day 10). Day 21 was set as the target quit day for cannabis (or before if participants were willing and able to). On week 12 maximum dose of nabiximols allowed was 21 sprays. (DOC) [file pone.0190768.s008.doc]

**S2 Table**

|  | **Week 1** | | | | | | |
| --- | --- | --- | --- | --- | --- | --- | --- |
| **Day** | **1** | **2** | 3 | 4 | 5 | 6 | 7 |
| **Maximal sprays** | **5** | **5** | 10 | 15 | 20 | 25 | 30 |

|  | **Week 2** | | | | | | |
| --- | --- | --- | --- | --- | --- | --- | --- |
| **Day** | 8 | 9 | **10** | 11 | 12 | 13 | 14 |
| **Maximal sprays** | 35 | 40 | **42** | 42 | 42 | 42 | 42 |

|  | **Week 3** | | | | | | | **Weeks 4 - 11** |
| --- | --- | --- | --- | --- | --- | --- | --- | --- |
| **Day** | 15 | 16 | 17 | 18 | 19 | 20 | **21** |
| **Maximal sprays** | 42 | 42 | 42 | 42 | 42 | 42 | **X** | **Quit at any moment**  **(42 sprays max.)** |

|  | **Week 12** | | | | | | | **End of Medication** |
| --- | --- | --- | --- | --- | --- | --- | --- | --- |
| **Day** | 78 | 79 | 80 | 81 | 82 | 83 | 84 |
| **Maximal sprays** | **21** | **21** | **21** | **21** | **21** | **21** | **21** |
