# Supplementary material for: Nabiximols combined with motivational enhancement/cognitive behavioral therapy for the treatment of cannabis dependence: A pilot randomized clinical trial
Source: PLoS One. 2018 Jan 31;13(1):e0190768. doi: 10.1371/journal.pone.0190768 (PMC5791962; doi:10.1371/journal.pone.0190768)
Supplement: S3 Table — Table represents concentrations in plasma specimens for Δ9-tetrahydrocannabinol (THC), 11-hydroxy-THC (11-OH-THC), 11-nor-9-carboxy-THC (THCCOOH), cannabidiol (CBD) and cannabinol (CBN), THC-glucuronide (THC-glu), THCCOOH-glucuronide (THCCOOH-glu) for nabiximols and placebo groups. (DOCX) [file pone.0190768.s009.docx]

|  | **Nabiximols Group** |  |  |  |  |
| --- | --- | --- | --- | --- | --- |
|  |  | **Week of Treatment** | | | |
|  |  | **0** | **4** | **8** | **12** |
| **Cannabinoids**  **(µg/L)** | **THC** | 4.8 | 4.2 | 1.7 | 4.5 |
|  | **11-OH-THC** | 1.3 | 2.2 | 0.9 | 1.4 |
|  | **THC-COOH** | 43.3 | 51.3 | 33.5 | 47.6 |
|  | **CBD** | 0.0 | 0.4 | 0.0 | 0.1 |
|  | **CBN** | 0.0 | 0.0 | 0.0 | 0.0 |
|  | **THC-glu** | 0.0 | 0.1 | 0.0 | 0.1 |
|  | **THCCOOH-glu** | 160 | 119 | 79.6 | 142 |

|  | **Placebo Group** |  |  |  |  |
| --- | --- | --- | --- | --- | --- |
|  |  | **Week of Treatment** | | | |
|  |  | **0** | **4** | **8** | **12** |
| **Cannabinoids (µg/L)** | **THC** | 8.2 | 3.0 | 2.6 | 2.4 |
|  | **11-OH-THC** | 3.2 | 1.7 | 1.8 | 1.4 |
|  | **THC-COOH** | 79.6 | 38.7 | 45.3 | 28.4 |
|  | **CBD** | 0.0 | 0.0 | 0.0 | 0.0 |
|  | **CBN** | 0.0 | 0.0 | 0.0 | 0.0 |
|  | **THC-glu** | 0.1 | 0.0 | 0.0 | 0.0 |
|  | **THCCOOH-glu** | 331 | 139 | 184 | 111 |
