# Supplementary material for: Nabiximols combined with motivational enhancement/cognitive behavioral therapy for the treatment of cannabis dependence: A pilot randomized clinical trial
Source: PLoS One. 2018 Jan 31;13(1):e0190768. doi: 10.1371/journal.pone.0190768 (PMC5791962; doi:10.1371/journal.pone.0190768)
Supplement: S1 File — (DOC) [file pone.0190768.s010.doc]

**Supporting Information**

**Urine specimen analysis**

Cannabinoids of interest in urine, including ∆^9^-tetrahydrocannabinol (THC), 11-hydroxy-THC (11-OH-THC), 11-nor-9-carboxy-THC (THCCOOH), cannabidiol (CBD) and cannabinol (CBN) were quantified with a fully validated two-dimensional gas chromatography-mass spectrometry method (2D-GCMS) as previously described . Instrument configuration was similar except for increasing the injection port temperature to 330°C to reduce matrix contamination and minor modifications to the oven program allowed inclusion of CBD (390.3, 458.3, 443.3), CBN (367.3, 382.3, 310.3) and d3-CBD (393.3, 461.3). Quantification ions are underlined. The initial oven temperature of 160°C was ramped at 20°C/min to 250°C and held for 4 min; followed by ramps of 75°C/min to 225°C (0.25 min hold), 15°C/min to 275°C (1.5 min hold), 75°C/min to 225°C (0.25 min hold) and 15°C/min to 275°C (2.7 min hold). A final ramp of 75°C/min to 320°C was held for 2.5 min to clean the column before re-equilibrating to the initial starting temperatures. Interface, ion source, and quadrupole temperatures were 280, 230, and 150°C, respectively. Similarly, modifications to the solid phase extraction (SPE) method also were necessary to incorporate the additional analytes. A sequential enzyme-alkaline tandem hydrolysis and ZSTHC020 columns (United Chemical Technology, Bristol PA) were utilized.

Briefly, 1 mL urine was combined with 25 µL working internal standard containing deuterated THC, 11-OH-THC, THCCOOH, and CBD analogs, 1 mL 0.1M phosphate buffer (pH 5.0), and 25 µL β-glucuronidase (Red Abalone) solution [100,000U/mL; Kura Biotec, Inglewood, CA]. Tubes were capped, vortexed and incubated at 37°C for 16 h in a shaking water bath. After cooling to room temperature, 80 μL 10N sodium hydroxide was added and tubes were capped, vortex mixed, and hydrolyzed for 20 min at 60°C. Concentrated glacial acetic acid (50 µL) was added after cooling to room temperature. Following tandem hydrolysis, proteins were precipitated with 2mL ice-cold acetonitrile; added in 0.5 mL aliquots, while vortexing. Tubes were centrifuged for 10 min at 1800 × *g* to pellet proteins. Supernatants were decanted into tubes containing 2 mL 2N sodium acetate buffer (pH 4.0) and vortex mixed. SPE columns were conditioned by sequential addition of 2 mL methanol, 2 mL deionized water, and 1 mL 0.1M phosphate buffer (pH 6.8). Supernatants were applied to conditioned SPE columns under low vacuum. Columns were washed with 3 mL deionized water, 3 mL 0.1M phosphate buffer (pH 6.8) and dried by full vacuum for 10 min. After priming the sorbent bed with 0.2 mL hexane, analytes were eluted with 5 mL elution solvent (hexane/ethyl acetate/glacial acetic acid 80:20:1, v/v/v) into 10-mL centrifuge tubes containing 0.5 mL ethanol. Eluates were evaporated to dryness under nitrogen at 40°C, reconstituted with 50 μL N,O-bis(trimethylsilyl) trifluoroacetamine with 1% trimethylchlorosilane (BSTFA-TMCS) and derivatized at 70°C for 30 min. 3 μL splitless injection was utilized for each sample. Linearity ranged from 2.5 to 250 μg/L (THC, 11-OH-THC, and CBD), 1.0–100 μg/L (CBN), and 5.0–500 μg/L (THCCOOH). Accuracy ranged from 91.2 to 111.0% for all analytes across the linear range. Intra- and inter-assay imprecision, as percent relative standard deviation was less than 10.8%. Limit of quantification (LOQ) values for the different cannabinoids were as follows: THC 2.5 µg/L, 11-OH-THC 2.5 µg/L, THCCOOH 5 µg/L, CBD 2.5 µg/L and CBN 2.5 µg/L. Urine concentrations for the cannabinoids of interest were normalized to creatinine and are represented as mean values.

**Plasma specimen analysis**

We quantified cannabinoids by a previously validated liquid chromatography–tandem mass spectrometry (LC-MS/MS) method by Schwope et al. . Briefly, we added 1.5 mL ice-cold acetonitrile drop wise to plasma (0.5 mL) while vortexing to precipitate proteins. After mixing, the sample was centrifuged and the supernatant diluted and subjected to SPE using Bond Elut Plexa 200mg/6 mL columns (Agilent, Santa Clara, CA). The eluent was evaporated and reconstituted in mobile phase. Extracts were chromatographed via gradient elution at 400 μL/min, and detection was via electrospray ionization. Linearity ranged from 0.5 to 50 μg/L (THC-glucuronide (THC-glu)), 1.0–100 μg/L (THC, 11-OH-THC, THCCOOH, CBD, and CBN), and 5.0–250 μg/L (THCCOOH-glucuronide (THCCOOH-glu)). Imprecision was <10.5% CV, and bias within ±13.1% of target for all analytes across the linear range. LOQ values for the different cannabinoids are as follows: THC 1 µg/L, 11-OH-THC 1 µg/L, THCCOOH 1 µg/L, THCCOOH-glu 0.5 µg/L, THC-glu 0.5 µg/L, CBD 1 µg/L and CBN 1 µg/L. Plasma values are represented as mean values (Figure S2).

**References**

1. Lowe RH, Karschner EL, Schwilke EW, Barnes AJ, Huestis MA. Simultaneous quantification of Delta9-tetrahydrocannabinol, 11-hydroxy-Delta9-tetrahydrocannabinol, and 11-nor-Delta9-tetrahydrocannabinol-9-carboxylic acid in human plasma using two-dimensional gas chromatography, cryofocusing, and electron impact-mass spectrometry. Journal of chromatography A. 2007;1163(1-2):318-27.

2. Schwope DM, Scheidweiler KB, Huestis MA. Direct quantification of cannabinoids and cannabinoid glucuronides in whole blood by liquid chromatography-tandem mass spectrometry. Analytical and bioanalytical chemistry. 2011;401(4):1273-83.

**SATIVEX ASSOCIATED WITH BEHAVIORAL-RELAPSE PREVENTION STRATEGY AS TREATMENT FOR CANNABIS DEPENDENCE**

Principal Investigator: Bernard Le Foll MD PhD

Translational Addiction Research Laboratory, CAMH

Co-Investigators: Peter Selby MBBS, MPH

Chief of Addictions, CAMH

Jurgen Rehm PhD

Director, Social and Epidemiological Research, CAMH

Marilyn Huestis PhD

Chief, Chemistry & Drug Metabolism, IRP, National Institute on Drug Abuse and Adjunct Professor, UMB School of Medicine

Benedikt Fischer PhD

Senior Scientist

Social, Prevention and Health Policy Research Department, CAMH

Lena Quilty PhD

Psychologist and Independent Scientist, Clinical Research, CAMH

Consulting Investigator: Tony George MD

Clinical Director, Schizophrenia, CAMH

**INTRODUCTION**

**Cannabis exposure, use disorders and treatment in North America**

Cannabis, referring to any number of preparations of the *Cannabis sativa* plant including marijuana, is the most widely used illicit substance worldwide . It is estimated that the number of people who had used cannabis at least once (aged 15 to 64) is between 129 and 191 million, or 2.9% to 4.3% of the world population, with North America the subregion with proportionally the largest user group with 30 million . Not surprisingly given these numbers, cannabis is the most widely used illicit drug in the United States . In 2008, the National Survey on Drug Use and Health (NSDUH) conducted by the Substance Abuse and Mental Health Services Administration (SAMHSA) estimated that 15.2 million Americans (aged 12+, approximately 6.1 % of the population) were current users (defined as past month usage) . Monthly prevalence has been consistently around 6.0% since 2002, with even higher usage reported among young adults (aged 18 to 25) at 16.5%. Among illicit drugs, cannabis also had the largest number of past year initiates (persons aged 12 or older) at 2.2 million . Canadian prevalence numbers are similarly high: surveys indicate that one in every ten adults had used cannabis in the last year, again with multifold higher prevalence (e.g., one in three in age group 16 - 25) in younger years . Cannabis use prevalence is important for public health , as it could be shown, that the number of problem users and cannabis-attributable problems depend on overall prevalence.

Research indicates that about 10% of those who ever use cannabis become regular daily users , which in turn is a strong risk factor for any kind of cannabis-attributable health problems including cannabis use disorders. In 2008, 4.2 million Americans had past year dependence or abuse, representing 1.7 percent of the total population aged 12+; this made up 60.1% of all illicit drug dependence or abuse. In 2007, 15.8% of people entering drug abuse treatment programs in the UWS reported cannabis as their primary drug of abuse (61% of those under 15), representing nearly 288,000 treatment admissions . In Canada, where the study will take place, treatment for problematic cannabis use consists mainly of cognitive behavioral interventions. This is similar to the common practice in the US and worldwide. Despite the fact that this approach is similar for other psychoactive and no novel treatments have been added, a consistently increasing number of individuals with cannabis problems have been admitted to treatment. Specifically, in the past decade, some 25 - 30% of admissions to publicly funded addiction treatment programs across Canada involved individuals with cannabis related problems . Specifically, in a study of all new admissions to publicly funded addiction treatment services in the fiscal year 2000/2001 in the province of Ontario, 14,633 cases, or 30.5% of total cases admissions (47,995) involved cannabis as a problem substance . Notably, these cannabis admissions involved individuals users considerably younger than for other substance use problems, 56% of cannabis admissions were <20 years of age . A sizeable share of admissions also consists of individuals entering treatment following coercion from the criminal justice system (e.g., by way of diversion programs, probation or parole conditions), and close to four in ten (38%) cannabis admissions featured a legal coercion background .

**Agonist Pharmacotherapy for drug dependence treatment**

Due to the large impact of cannabis on individuals and society, various research teams have explored the possibility of developing medications for cannabis dependence treatment. Various reviews are available on the topic . The main psychoactive ingredient in marijuana is Δ^9^-tetrahydrocannabinol (THC), but other substances such as cannabidiol could be modulating rewarding/reinforcing effects of THC. Two forms of cannabinoid receptors, CB_1_ and CB_2_, have been cloned . The CB_1_ receptor and its splice variant the CB_1_A receptor are predominantly found in the brain, with the highest density in the hippocampus, cerebellum, cortex and striatum, whereas the CB_2_ receptor is located peripherally, principally associated with the immune system . THC is partial agonist to the CB_1_, whereas cannabidiol is a CB_1/2_ antagonist . Interestingly, it has been suggested that THC and cannabidiol may have opposite properties: THC produces psychotic-like and anxiogenic effects in humans , whereas cannabidiol may have anti-psychotic and anxiolytic properties. Importantly, although cannabidiol has no reinforcing effects of its own , it can affect the ability of THC to induced conditioned place preference . Moreover, cannabidiol can enhance extinction of psychostimulant-induced place preferences and attenuate cue-induced opioid seeking behaviors in rats , suggesting that this ligand may have useful properties for drug dependence treatment.

The two main approaches in drug addiction treatment consist at either substituting the drug of abuse or at blocking the effects of the drug of abuse. However, due to the widespread and diverse roles of the endo-cannabinoid system, targeting one function of the system to produce beneficial effects commonly causes other functions to be adversely affected. There has been a lot of hopes in the use of antagonist at the cannabinoid CB_1_ receptor for treatment of drug dependence (see our review ). However, Rimonabant, the first clinically available member of this class of drugs, has been linked to increased risk of anxiety, depression, and suicidality and is no longer available . Therefore, substitution therapy is currently the approach that appears most promising. Since nicotinic agonists (nicotine replacement therapy and varenicline) are the most effective therapies for treatment of nicotine addiction and since opioid agonists (methadone and buprenorphine) are the most effective treatment for opiate dependence treatment, there are great hopes that cannabinoid agonists may be effective treatment for cannabis dependence.

In the last few years, there has been growing interest in the use of THC to reduce cannabis withdrawal and/or modulate self-administration behavior. In a laboratory settings, Haney *et al.* demonstrated that oral THC (50 mg/day) reduced ratings of withdrawal in volunteers, as compared to placebo . Budney *et al.* evaluated the efficacy of oral THC (30 and 90 mg/day vs placebo) on cannabis withdrawal symptoms . In this study, the highest dose was more effective than the lowest dose at decreasing withdrawal . Oral THC at the dose of 40 and 80 mg/day was also effective to decrease the subjective effects induced by smoking cannabis, but not to affect the choice to use cannabis . Similarly, 60 mg/day oral THC was not effective to reduce marijuana self-administration in a laboratory model of relapse . Although these two studies are not indicative of an efficacy of oral THC to attenuate self-administration behaviors, it should be noted that those studies were performed in non-treatment seekers and the exposure to oral THC was limited to few days. Therefore, it remains possible that administration of cannabinoid agonists in cannabis dependent subjects seeking treatment and for prolonged period of time may be effective. Indeed, case-reports support this view . However, to date, supporting evidence is still lacking. In August 2010, a search on the NIH Clinical trial Registry website found 3 clinical trials performed in treatment-seekers. The NCT00217971 study (PI Dr Levin) called ‘Dronabinol Treatment for Marijuana Addiction’ evaluated efficacy of oral THC for the treatment of cannabis dependence. The NCT00480441 study (PI Dr O’Brien) called ‘Effectiveness Study of Dronabinol and BRENDA for the Treatment of Cannabis Withdrawal’ focused on withdrawal symptoms and evaluated oral THC at the dose of 40 mg/day for 3 weeks combined with a behavioral intervention. The NCT01020019 study (PI Dr Levin) called ‘Combined Pharmacotherapy for Cannabis Dependency is starting in 2010 and will evaluate the efficacy of oral THC at the dose of 60 mg/day in association with lofexidine as treatment of cannabis dependence. Therefore, in spite of the evidence indicating that cannabidiol may be useful to curb drug addiction, no study has evaluated the efficacy of SATIVEX® (THC/cannabidiol combination in a buccal spray) administered for prolonged period of time in cannabis dependent subjects that are treatment seekers.

This project will take advantage of the approval within Canada of SATIVEX®. SATIVEX® is indicated in Canada as adjunctive treatment for the symptomatic relief of neuropathic pain in multiple sclerosis in adults. SATIVEX® is also indicated as adjunctive analgesic treatment in adult patients with advanced cancer who experience moderate to severe pain during the highest tolerated dose of strong opioid therapy for persistent background pain. Preclinical research indicates that THC and cannabidiol may have opposite properties (see above). Supporting this view, it has been recently reported that reinforcing effects of cannabis with high cannabidiol content is lower than cannabis with low cannabidiol content . Therefore, administering SATIVEX® may have therapeutic properties for treatment of cannabis dependence. However, to date, no study has evaluated the efficacy of SATIVEX® for treatment of cannabis dependence.

**OVERVIEW**

There will be first a pilot study in five subjects seeking treatment for cannabis dependence to ensure that our planned self-titration regimen is appropriate using SATIVEX® (THC/cannabidiol combination in a buccal spray). This phase will be open label, with no placebo control. The subjects will be treated using the same approach as outlined below for the RCT. Then, there will be a twelve-week, double-blind, placebo-controlled study in male and female subjects seeking treatment for cannabis dependence (n=40). Study visits will occur weekly during the medication phase of the study. The medication will be self-titrated over three weeks and a target quit date will be set up at Day 21. There will be a total of 12 weeks of drug exposure. Throughout these 12-weeks, all participants will receive a combination of pharmacotherapy (Sativex Spray or Placebo Spray) associated with a weekly intervention of combined Motivational Enhancement Therapy and Cognitive Behavioral Therapy (MET/CBT) in accordance with the intervention practices shown to be effective in treatment of cannabis dependence . The intervention will be adapted from the Brief Counseling for Marijuana Dependence manual published by the Substance Abuse and Mental Health Services Administration (SAMHSA). In addition, to ensure compliance a Contingency Management plan will be implemented with $5 voucher that will be provided at each visit that is attended and at which biological samples have been provided, but with 5$ voucher loss for a missed appointment. The vouchers will be provided at end of treatment phase and at end of the follow up phase.

At study visits, vital signs and self-report ratings will be collected. The subjects will have to come two times per week to the center to assess medication usage and will be asked to provide urine sample (two times weekly) and blood sample weekly during the treatment and follow-up phase and at the end of trial period (see Summary of Study Assessments). As there may be compliance issues, a contingency management approach will be also implemented.

Following the medication phase, participants will have a follow-up weekly for another four weeks and then monthly until the 6 month follow up visit after the treatment starting date. We are planning to enroll 45 subjects over the two-year period.

**Summary of Study Assessments**

| **Assessment** | **Baseline** | **Weekly**  **(Weeks 1-12)**  **medication phase** | **Weekly**  **(Weeks 13-16)** | **Monthly**  **(months 5-6)** |
| --- | --- | --- | --- | --- |
| Psychiatric Evaluation (including SCID) | X |  |  |  |
| Demographic | X |  |  |  |
| Bloodwork (CBC, SMA-12), Urinalysis, EKG | X |  |  | X (end of trial) |
| Physical Exam | X |  |  | X (end of trial) |
| Urine Toxicology | X | X | X | X |
| Vital Signs, Carbon Monoxide | X | X | X | X |
| Serum Pregnancy Test (Females only) | X | If needed | If needed | If needed |
| Urine Pregnancy Test (Females only) | X | X | X | X |
| FTND, Cigarette, alcohol and caffeine Time-line Follow-Back (TLFB) | X | X | X | X |
| Cannabis TLFB, Marijuana withdrawal checklist, MCQ | X | X | X | X |
| BDI, HAM-A, HAM-D, BPRS, Profile of Mood questionnaire, DEQ, SMHSQ | X | X | X | X |
| SAFTEE | X | X |  |  |
| ASI | X |  | One week after medication phase | X (end of trial) |
| Blood for THC metabolite analysis | X | X | X | X |
| Urine for THC metabolite analysis | X | X (two times per week) | X | X |

Note that for Urine analysis we will use a “double drop” urinalysis. Subjects will drink a large glass of water as they enter the clinic and then urinate (U1). 30 min later they urinate a second time (U2). Both samples will be analysed as normalized to creatinine levels, this would allow us to compare the variability of urinalysis results. As this is a pilot analysis, this measure will only be collected in limited number of subjects to a grand total of 100 samples.

Subjects will be screened initially during a telephone interview in which medical, psychiatric, and substance abuse histories will be obtained without personal identifiers. The qualified subjects will then be assessed using the Structured Clinical Interview for *DSM-IV* (SCID). The first 5 subjects will be treated open-label (non blinded) with SATIVEX®, and their urine and blood specimens will be transferred to Dr. Marilyn Huestis (NIH-NIDA-IRP) for initial analysis. The data collected with those first five subjects will be used to ensure that the self-titration schedule we have planned for the subsequent randomized clinical trial is appropriate. It is clear that based on the results of these five initial subjects, we could improve the design of the trial if deemed appropriate. Following the first five subjects, two groups of subjects will be randomized into a pilot clinical trial:

| **Experimental group** | **Experimental condition** |
| --- | --- |
| Group #1 **(n=20)** | Placebo Spray + MET/CBT |
| Group #2 **(n=20)** | SATIVEX® Spray + MET/CBT |


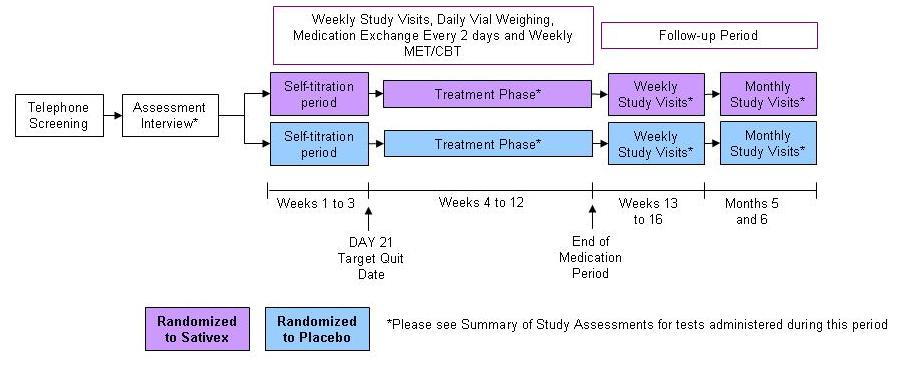


Weekly Study Visits, Vial Weighing, Medication Exchange and Weekly MET/CBT

***Primary and Secondary Outcome Measures***

For the main trial, our primary outcome measures 1) for tolerability will be the number of subjects that drop out because of SAEs in the two groups and 2) for piloting efficacy will be seven-day point prevalence cannabis abstinence one week after the end of medication phase and at the 6 month follow up (to allow for clear determination of abstinence status as there will be no Sativex exposure at those time points);

Secondary outcome measures will be 1) percentage of days of cannabis use over the study duration until the 6 month follow up; 2) amount of cannabis use over the study duration until the 6 month follow up. 3) effects on withdrawal symptoms scores, craving scores and number of urine/blood samples screen as positive.

We also will collect urine and blood samples to determine possibility of determining abstinence status based on those, but those studies will be independent of our main goals which are tolerability and efficacy.

**PRELIMINARY RESULTS**

A treatment group has been implemented at the Centre for Addiction and Mental Health for subjects with cannabis dependence treatment. So far, this treatment intervention has been delivered for one year. There is a waiting list with subjects interested to get into treatment at CAMH. Those preliminary results demonstrate the feasibility of recruiting the subjects for this study. We also have obtained the SATIVEX® Spray and Placebo Spray that will be needed for this project from GW Pharma (see letter enclosed). Dr Huestis from NIDA-IRP has also developed an innovative method allowing to detect cannabis abstinence is urine/blood sample of cannabis dependent subjects.

**METHODS:**

**Subjects**

*Inclusion criteria*

- Adult male or female (gender to be analyzed as a covariate)
- Understand and willing to comply with study requirements and restrictions
- Willing to use appropriate contraceptive method throughout the study
- Otherwise healthy as judged by investigator based on medical history, physical exam, vitals, ECG and labs
- DSM-IV criteria for current marijuana dependence
- Report marijuana as primary drug of abuse
- Report using marijuana at least 5 days a week for at least one month
- Have marijuana positive urine drug screen
- Treatment seeking cannabis smoker
- Smoke less than or equal to the equivalent of 4 joints per day (or four grams per day if participants smokes cannabis in other forms)

*Exclusion criteria*

- Meets DSM-IV criteria for a current axis I disorder including substance use disorder other than cannabis, nicotine or caffeine dependence.
- First-degree relative with schizophrenia
- History of seizures
- History of cardiovascular disease
- History of pulmonary disease such as asthma, COPD
- Clinically significant pathology in oral cavity and poor oral hygiene
- Known sensitivity to dronabinol, cannabidiol, propylene glycole, ethanol or peppermint oil (used in SATIVEX® buccal spray)
- Unstable medical conditions
- Pregnant or breast-feeding
- Currently taking psychotropic medication with benefit for any other illness than treatment of insomnia,
- Holding a job that involves driving, operating heavy machines

*Pre-study Screening and Baseline/follow up evaluations and questionnaires that will be used:*

a) Structured Clinical Interview for DSM-IV Axis I Disorders (SCID-I)

b) Demographic assessments

c) Psychiatric/Medical Evaluation and Physical Examination by study physician Weight (kg) and Vital Signs (Temperature, Pulse, Blood Pressure and Respiration Rate). Carbon monoxide will be taken at each study visit to assess exposure to smoke (will reflect both cannabis and tobacco exposure)

d) 12-lead Electrocardiogram (EKG).

1. Blood work, including complete blood count (CBC), electrolytes, renal and liver function tests
2. For female subjects, a serum pregnancy test (beta-HCG) at inclusion (and when deemed needed) and weekly urine pregnancy test will be performed. Female subjects will be asked if they are lactating.

g) Ten-panel urine toxicology screen at baseline, and at each study visit to ensure absence of illicit drug use

h) Substance use assessment including the age of first use, age of regular use,

number of days in the past 30 days that the substance was used, number of days in the past week that the substance use used, and the amount of the substance used per using day (i.e. joints per day for cannabis)

i) Cannabis Use will be assessed by toxicology and self-report. Participants will provide a urine sample two times per week and a blood sample once weekly (see assessment schedule). Samples will be frozen and sent to Dr Huestis at NIDA-IRP in order to analyze those samples using her novel method of detection of abstinence in cannabis users using biological samples. The timeline follow-back (TLFB) will be used to assessing the number of days cannabis was smoked, the amount smoked, and the amount of money spent on cannabis since their last appointment. Smoking diaries will also be completed daily outlining how much cannabis was obtained at a time, how many days that amount lasted, and what amount, if any, was shared. This diary will also record number of sprays delivered each day.

j) Addiction Severity Index

k) Cannabis Withdrawal will be assessed using the Marijuana Withdrawal Checklist with 16 4-point items indicating severity during the prior 24 hours . Cannabis craving will be assessed using the *Cannabis Craving Questionnaire (MCQ) .*

l) The sleep disturbance will be assessed using the St Mary’s Hospital Sleep Questionnaire (SMHSQ), a self-report instrument developed for use with psychiatric and medical in-patients .

m) The Hamilton Anxiety Scale (HAM-A), will be used to assess the affective and vegetative symptoms of anxiety .

n) Depressive symptoms will be assessed using the Beck Depression Inventory (BDI - a self-report depression rating scale has been used extensively in depression treatment research ) and the Hamilton Depression Rating Scale (HDRS), 21-item version, a clinical administered rating scale for depression with well-established reliability and validity ; The Profile of Mood States will also be used.

o) Psychotic symptoms will be assessed using the Brief Psychiatric Rating Scale (BPRS)

p) Cigarette smoking history including timeline follow-back (TLFB) for the past one week ; Pack-Years (average daily smoking consumption in packs per day multiplied by the years smoked); Fagerstrom Test for Nicotine Dependence (FTND).

q) Alcohol and caffeinated beverage use TLFB for the past one week .

r) The SAFTEE will be used to monitor for treatment-emergent medication side effects.

s) During the abstinence condition, participants will complete the Drug Effects Questionnaire (DEQ). The DEQ has eight items (drug effect, bad effect, good effect, liked, sick, sedated, stimulated and want to take again) measured on a 100 mm visual analog scale.

**Pharmacological Treatment: SATIVEX® buccal Spray (Active and Placebo)**

1. Pilot Study in 5 subjects:

Although SATIVEX® has been tried up to 48 Spray per day in human subjects (Personal information from Dr Robson, Scientific Director of GW Pharma), there is currently limited information to guide us on the SATIVEX® dosage to use in cannabis dependent individuals that likely have developed strong tolerance to cannabinoids. Therefore and since there is a large inter-individual variability between subjects in the quantity of cannabis smoked, we are planning to use a self-titration approach. The first five subjects will be treatment-seekers that fit our inclusion/exclusion criteria and that will be treated open-label. These subjects will be instructed to use the SATIVEX® Spray according to the induction schedule provided below. The first few sprays will be administered in front of medical staff, so we can ensure proper use and good tolerability. This first subjects will allow us to determine if our schedule for dosing is appropriate for the subsequent phase of the study.

2) Pilot Double Blind trial in 40 subjects:

Study subjects (n=40) will be randomized in blocks of 10 to one of the two groups (Sativex vs. placebo) in a 1:1 ratio and in a double blind manner. There will be an induction phase for two weeks, then one week to get to steady state. During the first day of medication administration, dose administration will be supervised and subjects will be monitored for 2 hours to ensure tolerability. During this period, the Drug Effects Questionnaire will be administered every 30 minutes to assess initial effects of medication on participants. SATIVEX® will be self-titrated. There will be a gradual increase of the maximal allowed dose starting at five sprays per day for the first two days and increasing of five sprays per day until reaching the max number of 42 sprays maximal per day at end of week 2 (Day 11). The target quit date will be set at Day 21 (but subjects will be allowed to stop using cannabis before if they are willing and able to). The medication treatment phase will then be continued for an additional 9 weeks (the last week will be a reduction phase with the use of Spray that will be decreased by 50% to avoid abrupt withdrawal). A set of instructions for SATIVEX use will be provided to the subjects. Then exposure to medications will be stopped (so there will be a total of 12 weeks medication treatment exposure). Subsequently there will be weekly visit for four weeks and then follow up visit monthly

A dose of 48 sprays per day has been shown to be tolerable in human studies. Our current dose of 42 sprays per day is within the dosage range observed to be tolerable. It should be noted that we are not expecting that this highest dose will be used by most of the study participants. Moreover, we expect that this dosage will be well tolerated as the subjects have developed tolerance to cannabinoids and also will self-titrate allowing adjusting their intake based on tolerability. Several steps have been taken to ensure safety of subjects participants. For safety reasons and to avoid diversion, subjects will come two times per week during week days at CAMH to assess their usage of SATIVEX®. This will be completed by weighing the used vials. SATIVEX® vials will be exchanged during study visits at CAMH.

**Biological sample preparation (blood)**

Blood samples will be collected in a grey top vacutainer tube (fluoride tube) for THC and metabolite determination. Once samples are collected, they are to be placed on ice allowed to clot fully and then spun and separated. This should be conducted as soon as possible after the aforementioned requirements have been met, but should not exceed 2 hours since samples collection. Plasma aliquots should be stored at -20°C until sample shipment.

**Biological sample collection (excludes samples used for general health assessments)**

Urine samples will be collected two times a week and blood samples collected weekly. All participants will have urine and blood sample collected on the weekly visit. The second urine sample will also be collected during MET/CBT visits. At any point that biological samples are taken, information about the last time the participant may have smoked cannabis and last used the study medication will be collected. All urine samples will be taken at the beginning of the visit whereas blood samples will be collected at the completion of the study visit to standardize the time since last spray. Each urine sample obtained will have creatinine measures determined by the CAMH laboratory which will be required for THC quantification by Dr. Huestis at NIDA. Ten (10ml) of urine and plasma samples will be sent to NIDA every 2 months for quantification and will be stored at -20°C. Shipment of samples must follow Transportation of Dangerous Goods guidelines. Appropriate temperature control during shipment should be maintained.

**Biological sample collection for general health assessments**

Urine and blood samples for general health assessments will be collected at Baseline and at the end of trial (see Summary of Study Assessments).

**Psychological support: MET/CBT intervention**

All subjects will receive a weekly MET/CBT session with a trained therapist. This is an adaptation of a nine-week MET/CBT intervention which has been previously studied for the treatment of cannabis dependence and has been found to be effective . The intervention in the proposed study emphasizes the development of motivation for change and the implementation of skills to reduce and abstain from cannabis use, using the Brief Counseling for Marijuana Dependence manual published by the Substance Abuse and Mental Health Services Administration (SAMHSA). In this intervention, use of empathic therapeutic style helps resolve ambivalence, increase discrepancies about personal goals and cannabis use, and elicit motivation to change. Once ambivalence about cannabis use is addressed, the sessions focus on the cognitive, behavioral and emotional changes necessary for dealing with cannabis dependence while supplying coping strategies for predictable obstacles. As studies of psychosocial interventions in cannabis dependence have shown that more intensive interventions have a more sustainable outcome , the MET/CBT sessions in the proposed trial will continue for the full 12 weeks of the medication phase. The current SAMHSA manual provides an outline for this intervention for 9 weeks and provides 4 additional elective topics. In this study, participants will receive the 9 weekly sessions as outlined and then provided 3 of these elective topics to maintain the 12 week intervention length. These elective topics will be identical amongst all participants. Addition of this intervention will allow for the necessary conditions to assess the additive value of treatment with SATIVEX® for cannabis dependence.

**Recruitment:**

Participants will be mainly recruited from individuals seeking treatment within the CAMH Addiction Program. Information about this study will be presented to individuals through a brochure that will be present within the clinics or potentially by a clinician directly to their patients. After reviewing the information in the brochure, interested parties can contact the study coordinator through the telephone number provided in this brochure to obtain further detailed information about the study. Subjects will be also recruited from other addiction clinics or medical centers located in Toronto, by word of mouth, smoke paraphernalia shops, local advertisement in Toronto newspapers and through posters distributed in and around the CAMH and the University of Toronto campuses and on the CAMH “find a study” registry. Ads will also be posted online on U of T bulletins, Craigslist and similar websites, local magazines such as NOW! Or Eye Weekly and other mass media as radio or tv may also be used to recruit prospective subjects. Ads and fliers will be will approved by CAMH’s Research Ethics Board (REB). Subject eligibility will be initially determined through a telephone interview prescreening to assess potential eligibility.

**Participant Compensation:**

Once participants are deemed eligible to be enrolled in the study (following baseline assessments) the total monetary compensation provided to participants in this study will be $700. They will be provided $200 upon completion of the first 4 weeks of the trial, $200 after completion of the 12 week medication phase and $300 after completion of the 6 month follow-up period.

Due to the frequent visits required to CAMH, participants will also be provided with TTC tokens (or its cash value if preferred by participants) during treatment and follow-up visits as required.

*Contingency Management*

Participants will be provided with a $5 voucher for each visit where a sample of urine or blood is collected, however for each missed appointment, a $5 value will be subtracted from the total amount. These vouchers will be provided at two separate times, after completion of the treatment phase of the study, and after completion of the follow-up period.

In order to engage participants and to maximize returns to CAMH for daily visits, a “fish-bowl prize” system will be instituted where during each visit participants will have a chance to reach within a large bowl/drum and select a ticket which provides a chance to win a prize. These prizes will range from tickets containing motivational messages, a small prize such as a pen or notepad, a $5 gift card to Tim Hortons up to a to a maximum prize of $50 in the form of a gift card to a number of establishments including restaurants, grocery stores, or retail merchants.

**Data analysis:** All analyses will be implemented in SAS v.9.1.3 (The SAS Institute, Cary, NC). Associations with p-values of less than 0.05 will be considered statistically significant, and all tests will be 2-sided. The data will be screened prior to analysis to ensure that the underlying assumptions for all subsequent statistical procedures are met. Preliminary analyses will include chi-square tests, t-tests, and Mann-Whitney U tests.

Our Primary outcome measures will be: 1) tolerability which will be assessed by determining the number of participants who withdraw from the study because of SAEs and 2) pilot efficacy of this intervention will be assessed by assessing seven-day point prevalence cannabis abstinence one week after the end of medication phase; and at 6 month follow up. Secondary outcome measures will be: 1) percentage of days of cannabis use over the study duration until the at 6 month follow up; 2) amount of cannabis use over the study duration until the 6 month follow up and 3) effects on withdrawal symptoms scores, craving scores and number of urine/blood samples screen as positive. Efficacy analyses of count variables (e.g. number of joints per week) will be performed using generalized estimating equations (GEEs), specifying a negative-binomial distribution for the counts. Linear mixed models will be used to analyze continuous, secondary outcome measures (e.g. mood and anxiety ratings, physiological measures). All efficacy analyses will be by intention-to-treat. The intervention group will be treated as a between-subjects effect in each of these models, treatment week will measure changes within-subjects, and the interaction between intervention group and treatment week will be investigated.

Subjects will be considered abstinent based on self-report TLFB. As THC is a component of SATIVEX®, THC metabolites are naturally expected to be present in the urine for both cannabis-using participants as well as abstinent participants using SATIVEX®. Thus, standard urine sampling for THC metabolites cannot be easily used as a measure of abstinence in this study. To address this, our collaborator Dr. Marilyn Huestis (NIH-NIDA-IRP) will apply a novel modeling method on the results obtained with cannabinoid metabolite in biological sample in an attempt to use quantitative measures to determine use of SATIVEX® alone vs cannabis smoking. Urine samples will be collected two times per week and blood will be collected once a week (during the duration of the study) and will be sent to NIDA-IRP for analysis of THC metabolites.

**Power calculation:** The goal is to collect pilot data to demonstrate tolerability and trend for efficacy of SATIVEX® for treatment of cannabis dependence. We are hypothesizing that the use of cannabis in the group treated with SATIVEX® will decrease by at least 50% and that there will be increased rates of abstinence in the group receiving SATIVEX®, as compared to placebo spray. With a sample of 18 subjects per group (2 subjects loss during follow up and assuming an abstinence rate of 10% in the control group), we will have sufficient power to detect a difference in abstinence rates across the two study groups if the proportion of subjects who are abstinent in the study group is 50% or higher (based on Chi-square or logistic regression analysis). If more subjects are dropping out than expected, we will replace subjects and modify our contingency management plan to enhance compliance. Based on this calculation, we will be underpowered. Our goal with this pilot study is to determine the number of subjects that will be needed to validate this approach in a large sample.

**Termination of the Study**

Reasons for withdrawing individual subjects from the study may include one or more of the following:

1. Severe side effects;
2. Major protocol violation;
3. Subject lost to follow-up;
4. Withdrawal of consent;
5. Pregnancy

Any subject may be discontinued from the study at the discretion of the investigators if it is deemed to be in the best interest of the subject.

**Study Documentation**

Investigators will retain a subject identification code list if they need to contact subjects after the study. This list will contain the complete name, identification number, address and phone number of all subjects and will be held confidentially at the investigators site after completion of the study.

A CRF will be completed for each subject enrolled in the study. A subject screening log, noting reasons for screen failure, where applicable, will be maintained for all subjects. All information recorded on the CRFs for this study will be consistent with the subject’s source documentation (e.g. medical records). The investigator will document the obtained informed consent and record all medication administration, medical history, results of laboratory tests and adverse events in the CRF. Laboratory printouts and psychometric assessments will be considered source documents and will be incorporated into the CRF without transcription in a confidential manner.

The data collected during the study will be typed or written onto the CRF and other study documents using a ballpoint pen. If an error is made, it will be crossed out with a single horizontal line, and the new information clearly recorded next to the error, initialed and dated. Correction fluid is not allowed at any time.

**Archiving of Study Documentation**

Study data and other essential documents will be retained in a secure setting by the investigators for a period of 25 years as required by provincial regulations.

**Confidentiality**

All personal study participant data collected and processed for the purposes of this study will be managed by the investigators and their research staff with adequate precautions to ensure the confidentiality of this data, and in accordance with applicable national and local laws and regulations on personal data protection. The ethics committees approving this research and Health Canada will be granted direct access to the study participant’s original medical records for verification of clinical trial procedures and/or data, without violating the confidentiality of the subjects, to the extent permitted by the law and regulations. In any presentations of the results of this study at meetings or in publications, the subject’s identity will remain confidential.

**Adverse Events Reporting**

In the event of a serious adverse event (SAE), whether related to study drug or not, the Principal / Qualified investigator will notify the study Data Safety Monitoring Board, REB and NIDA of it occurrence and will submit the necessary documentation to their attention within timelines provided in CAMH SOP HSR207 Serious Adverse Event (SAE) Reporting. Only SAEs that are Suspected Unexpected Serious Adverse Reactions will be reported to Health Canada as outlined in ICH E2A Clinical Safety Data Management Definitions and Standards for Expediting Reporting.

While only SAEs and SUSARs will be reported as documented above, every adverse event will be assessed and recorded in the subject’s research record by research staff working on the study and reviewed regularly by the Principal/Qualified Investigator. Reporting of AEs will occur to the DSMB through Data Safety Monitoring Reports completed prior to DSMB meetings and to the REB in the annual renewal document.

The trial will be conducted in compliance with protocol, GCP, and applicable regulatory requirements.

REFERENCES:

1. Lowe RH, Karschner EL, Schwilke EW, Barnes AJ, Huestis MA. Simultaneous quantification of Delta9-tetrahydrocannabinol, 11-hydroxy-Delta9-tetrahydrocannabinol, and 11-nor-Delta9-tetrahydrocannabinol-9-carboxylic acid in human plasma using two-dimensional gas chromatography, cryofocusing, and electron impact-mass spectrometry. Journal of chromatography A. 2007;1163(1-2):318-27.

2. Schwope DM, Scheidweiler KB, Huestis MA. Direct quantification of cannabinoids and cannabinoid glucuronides in whole blood by liquid chromatography-tandem mass spectrometry. Analytical and bioanalytical chemistry. 2011;401(4):1273-83.

3. United Nations Office on Drugs and Crime. Vienna, Austria: 2010.

4. Administration SAaMHS. Results from the 2008 National Survey on Drug Use and Health: National Findings. Rockville, MD: Department of Health and Human Services, 2009.

5. Adlaf EM, Begin P, Sawka E. Canadian Addiction Survey (CAS): a national survey of Canadians' use of alcohol and other drugs: prevalence of use and related harms - a detailed report. Ottawa, ON: . 2005.

6. Health Canada. Canadian Alcohol and Drug Use Monitoring Survey (CADUMS): Summary Results for 2008. 2009.

7. Fischer B, Rehm J, Hall W. Cannabis use in Canada - The need for a public health approach. Canadian Journal of Public Health. 2009;100:101-3.

8. Room R, Fischer B, Hall W, Lenton S, Reuter P, editors. Cannabis policy: Moving beyond stalemate. New York, NY: Oxford University Press; 2010.

9. Hall W, Pacula R. Cannabis use and dependence: Public health and public policy. Melbourne: Cambridge University Press.; 2003.

10. National Institute on Drug Abuse. Marijuana. National Institute on Drug Abuse, 2010 may 2010. Report No.

11. Urbanoski K, Strike C, Rush BR. Individuals seeking treatment for cannabis-related problems in Ontario: demographic and clinical profile. European Addiction Research. 2005;11:115-23.

12. Rush B, Urbanoski K. Estimating the demand for treatment for cannabis-related problems in Canada. International Journal of Mental Health and Addiction. 2007;5:181-6.

13. Nordstrom BR, Levin FR. Treatment of cannabis use disorders: a review of the literature. Am J Addict. 2007;16(5):331-42.

14. Vandrey R, Haney M. Pharmacotherapy for cannabis dependence: how close are we? CNS Drugs. 2009;23(7):543-53.

15. Budney AJ, Roffman R, Stephens RS, Walker D. Marijuana dependence and its treatment. Addict Sci Clin Pract. 2007;4(1):4-16.

16. Elkashef A, Vocci F, Huestis M, Haney M, Budney A, Gruber A, et al. Marijuana neurobiology and treatment. Subst Abus. 2008;29(3):17-29.

17. Matsuda LA, Lolait SJ, Brownstein MJ, Young AC, Bonner TI. Structure of a cannabinoid receptor and functional expression of the cloned cDNA. Nature. 1990;346(6284):561-4.

18. Gerard CM, Mollereau C, Vassart G, Parmentier M. Molecular cloning of a human cannabinoid receptor which is also expressed in testis. Biochem J. 1991;279 ( Pt 1):129-34.

19. Munro S, Thomas KL, Abu-Shaar M. Molecular characterization of a peripheral receptor for cannabinoids. Nature. 1993;365(6441):61-5.

20. Howlett AC, Barth F, Bonner TI, Cabral G, Casellas P, Devane WA, et al. International Union of Pharmacology. XXVII. Classification of cannabinoid receptors. Pharmacol Rev. 2002;54(2):161-202.

21. Pertwee RG. Ligands that target cannabinoid receptors in the brain: from THC to anandamide and beyond. Addict Biol. 2008;13(2):147-59.

22. D'Souza DC, Abi-Saab WM, Madonick S, Forselius-Bielen K, Doersch A, Braley G, et al. Delta-9-tetrahydrocannabinol effects in schizophrenia: implications for cognition, psychosis, and addiction. Biol Psychiatry. 2005;57(6):594-608.

23. D'Souza DC, Perry E, MacDougall L, Ammerman Y, Cooper T, Wu YT, et al. The psychotomimetic effects of intravenous delta-9-tetrahydrocannabinol in healthy individuals: implications for psychosis. Neuropsychopharmacology. 2004;29(8):1558-72.

24. D'Souza DC, Ranganathan M, Braley G, Gueorguieva R, Zimolo Z, Cooper T, et al. Blunted psychotomimetic and amnestic effects of delta-9-tetrahydrocannabinol in frequent users of cannabis. Neuropsychopharmacology. 2008;33(10):2505-16.

25. Zuardi AW, Crippa JA, Hallak JE, Moreira FA, Guimaraes FS. Cannabidiol, a Cannabis sativa constituent, as an antipsychotic drug. Braz J Med Biol Res. 2006;39(4):421-9.

26. Guimaraes FS, Chiaretti TM, Graeff FG, Zuardi AW. Antianxiety effect of cannabidiol in the elevated plus-maze. Psychopharmacology (Berl). 1990;100(4):558-9.

27. Vann RE, Gamage TF, Warner JA, Marshall EM, Taylor NL, Martin BR, et al. Divergent effects of cannabidiol on the discriminative stimulus and place conditioning effects of Delta(9)-tetrahydrocannabinol. Drug Alcohol Depend. 2008;94(1-3):191-8.

28. Parker LA, Burton P, Sorge RE, Yakiwchuk C, Mechoulam R. Effect of low doses of delta9-tetrahydrocannabinol and cannabidiol on the extinction of cocaine-induced and amphetamine-induced conditioned place preference learning in rats. Psychopharmacology (Berl). 2004;175(3):360-6.

29. Ren Y, Whittard J, Higuera-Matas A, Morris CV, Hurd YL. Cannabidiol, a nonpsychotropic component of cannabis, inhibits cue-induced heroin seeking and normalizes discrete mesolimbic neuronal disturbances. J Neurosci. 2009;29(47):14764-9.

30. Le Foll B, Goldberg SR. Cannabinoid CB_1_ receptor antagonists as promising new medications for drug dependence. J Pharmacol Exp Ther. 2005;312(3):875-83.

31. Le Foll B, Gorelick DA, Goldberg SR. The future of endocannabinoid-oriented clinical research after CB1 antagonists Psychopharmacology (Berl). 2009;205:171-4.

32. Haney M, Hart CL, Vosburg SK, Nasser J, Bennett A, Zubaran C, et al. Marijuana withdrawal in humans: effects of oral THC or divalproex. Neuropsychopharmacology. 2004;29(1):158-70.

33. Budney AJ, Vandrey RG, Hughes JR, Moore BA, Bahrenburg B. Oral delta-9-tetrahydrocannabinol suppresses cannabis withdrawal symptoms. Drug Alcohol Depend. 2007;86(1):22-9.

34. Hart CL, Haney M, Ward AS, Fischman MW, Foltin RW. Effects of oral THC maintenance on smoked marijuana self-administration. Drug Alcohol Depend. 2002;67(3):301-9.

35. Haney M, Hart CL, Vosburg SK, Comer SD, Reed SC, Foltin RW. Effects of THC and lofexidine in a human laboratory model of marijuana withdrawal and relapse. Psychopharmacology (Berl). 2008;197(1):157-68.

36. Levin FR, Kleber HD. Use of dronabinol for cannabis dependence: two case reports and review. Am J Addict. 2008;17(2):161-4.

37. Morgan CJ, Freeman TP, Schafer GL, Curran HV. Cannabidiol attenuates the appetitive effects of Delta 9-tetrahydrocannabinol in humans smoking their chosen cannabis. Neuropsychopharmacology.35(9):1879-85.

38. Budney AJ, Novy PL, Hughes JR. Marijuana withdrawal among adults seeking treatment for marijuana dependence. Addiction. 1999;94(9):1311-22.

39. Heishman SJ, Singleton EG, Liguori A. Marijuana Craving Questionnaire: development and initial validation of a self-report instrument. Addiction. 2001;96(7):1023-34.

40. Ellis BW, Johns MW, Lancaster R, Raptopoulos P, Angelopoulos N, Priest RG. The St. Mary's Hospital sleep questionnaire: a study of reliability. Sleep. 1981;4(1):93-7.

41. Hamilton M. The assessment of anxiety states by rating. Br J Med Psychol. 1959;32(1):50-5.

42. Beck AT, Steer RA. Beck Depression Inventory. Philadelphia, PA, Center for Cognitive Therapy. 1987.

43. Hamilton M. A rating scale for depression. J Neurol Neurosurg Psychiatry. 1960;23:56-62.

44. McNair DM, Lorr M, Droppleman LF. Manual for the profile of mood states. San Diego, CA: Educational and Industrial Testing Services; 1971.

45. Sobell LC, Sobell MB, Leo GI, Cancilla A. Reliability of a timeline method: assessing normal drinkers' reports of recent drinking and a comparative evaluation across several populations. Br J Addict. 1988;83(4):393-402.

43. Heishman SJ, Singleton EG, Liguori A: **Marijuana Craving Questionnaire:**

**development and initial validation of a self-report instrument**. *Addiction* 2001, **96**(7)

:1023-1034.

**Minimal Anonymized Data Set**

| **SubjectID** | **Gender** | **Age** | **Ethnicity** | **Education Level** | **Employment Status** | **Marriage Status** |
| --- | --- | --- | --- | --- | --- | --- |
|  |  |  |  |  |  |  |
| 008 | Male | 49 | White: North American | Graduate Degree | Self-employed | Divorced/Separated |
| 009 | Male | 28 | Caucasian; White: European; White: North American | College | Work Odd Jobs; Self-Employed | Single (never married) |
| 010 | Male | 54 | White: North American | College | Welfare | Divorced/Separated |
| 011 | Male | 26 | Indian: Caribbean (E. Indian) | High School | Work Odd Jobs | Single (never married) |
| 012 | Male | 28 | White: European; White: North American | Trade School | Part-time; Self-Employed; Student; Work Odd Jobs | Single (never married) |
| 013 | Male | 26 | Indian: Caribbean (E. Indian) | High School | Work Odd Jobs | Single (never married) |
| 014 | Male | 23 | Asian: east | High School | Student | Single (never married) |
| 015 | Male | 64 | Caucasian | Part of High School | Retired | Widowed |
| 016 | Male | 20 | Asian: East | University | Student | Single (never married) |
| 017 | Male | 37 | Black: Caribbean; White: North American | High School | Welfare | Married/Common-law/same-sex Partner |
| 019 | Female | 47 | White: North American | Part of High School | Welfare | Divorced/Separated |
| 020 | Female | 23 | Caucasian | Part of High School | Homemaker | Married/Common-law/same-sex Partner |
| 021 | Male | 25 | Caucasian | University | Full-time | Single (never married) |
| 022 | Male | 27 | White: European | University | Student | Married/Common-law/same-sex Partner |
| 023 | Female | 55 | White: North American | High School | Full-time | Divorced/Separated |
| 024 | Male | 34 | White: North American | College | Student | Single (never married) |
| 025 | Female | 22 | Caucasian | University | Part-time | Single (never married) |
| 027 | Female | 37 | Asian: South | University | Full-time | Married/Common-law/same-sex Partner |
| 028 | Male | 59 | Caucasian | University | Not Employed | Single (never married) |
| 029 | Male | 27 | Mixed Background | Graduate Degree | Self-employed | Married/Common-law/same-sex Partner |
| 030 | Male | 41 | White: North American | Part of High School | Welfare | Single (never married) |
| 031 | Female | 29 | Caucasian | College | Part-time | Single (never married) |
| 032 | Male | 60 | White: European; Other | University | Work Odd Jobs; Retired | Divorced/Separated |
| 033 | Male | 25 | White: North American | College | Student; Part-time | Single (never married) |
| 035 | Male | 24 | Black: African; Black: North American | | Student | Single (never married) |
| 036 | Female | 36 | White: European | college | Full-time | Single (never married) |
| 037 | Male | 26 | Caucasian | college | Part-time | Single (never married) |
| 038 | Male | 22 | Black: North American | college | Not Employed | Single (never married) |
| 039 | Female | 28 | Caucasian; Middle Eastern | University | Part-time;Work Odd Jobs | Single (never married) |
| 040 | Female | 38 | Asian: East | University | Not Employed | Single (never married) |
| 041 | Male | 24 | White: European | High School | Part-time | Single (never married) |
| 042 | Male | 21 | Mixed Background | High School | Student | Single (never married) |
| 044 | Male | 25 | Caucasian; Hispanic | College; Advanced Diploma: Sustainable Energy and Building Technology | Self-employed | Married/Common-law/same-sex Partner |
| 045 | Female | 37 | Hispanic; Latin American | University | Part-timw | Single (never married) |
| 047 | Male | 28 | Asian: South East | High School | Part-time/ Student | Single (never married) |
| 048 | Female | 26 | White: North American | University | Full-time | Married/Common-law/same-sex Partner |
| 049 | Male | 35 | Caucasian | college | Part-time | Single (never married) |
| 050 | Male | 25 | Mixed:Background | college | Student | Single (never married) |
| 051 | Male | 33 | White: European | College | Self-employed | Single (never married) |
| 053 | Male | 26 | Caucasian | College | Self-employed | Single (never married) |

**Good Tolerability:** N# of SAE

|  | **Nabiximols # of Participants That Withdrew Due SAE** | **Placebo # of Participants That Withdrew Due SAE** |  |
| --- | --- | --- | --- |
| **Participants Analyzed** [Units: Participants] | **20** | **20** |  |
| **Tolerability** [Units: Participants] Count of Participants | **0** | **0** |  |

**Decreased cannabis use**

| **Nabiximols** | | | |  | **Placebo** | | | |
| --- | --- | --- | --- | --- | --- | --- | --- | --- |
|  |  |  |  |  |  |  |  |  |
| **BASELINE** | |  | **WEEK 12** |  | **BASELINE** | |  | **WEEK 12** |
|  |  |  |  |  |  |  |  |  |
| **ID** | **grams** |  | **grams** |  | **ID** | **grams** |  | **grams** |
| **10** | 14 |  | 0.0 |  | **8** | 6.2 |  | 0 |
| **12** | 2.75 |  | 0.0 |  | **9** |  |  |  |
| **14** | 2 |  | 0.0 |  | **11** | 5.75 |  | 9.6 |
| **16** |  |  |  |  | **13** | 5.25 |  | 1.4 |
| **17** | 8.5 |  | 10.0 |  | **15** |  |  |  |
| **20** |  |  |  |  | **19** | 5.25 |  | 3.67 |
| **21** | 2 |  | 0.0 |  | **23** | 5 |  | 0 |
| **22** | 2.25 |  | 1.3 |  | **27** | 5.25 |  | 4.82 |
| **24** | 4.6 |  | 0.0 |  | **28** | 2.625 |  | 0 |
| **25** | 7 |  | 2.7 |  | **29** |  |  |  |
| **31** |  |  |  |  | **30** | 15 |  | 12.25 |
| **32** | 7 |  | 5.0 |  | **33** | 0.6875 |  | 0 |
| **35** |  |  |  |  | **36** | 6 |  | 0 |
| **38** |  |  |  |  | **37** |  |  |  |
| **39** | 3.5 |  | 0.0 |  | **41** | 8.75 |  | 0 |
| **40** | 0.875 |  | 0.5 |  | **42** | 0 |  | 7 |
| **44** |  |  |  |  | **45** |  |  |  |
| **47** |  |  |  |  | **48** | 7 |  | 4.5 |
| **49** | 3.5 |  | 2.5 |  | **50** |  |  |  |
| **51** | 21 |  | 2.0 |  | **53** | 2.75 |  | 0 |
|  |  |  |  |  |  |  |  |  |
| **Average** | **6.1** |  | **1.8** |  |  | **5.4** |  | **3.1** |

**No increase in craving or withdrawal**

Please see attached excel file
